# Straightforward synthesis of *N*-arylindoles *via* one-pot Fischer indolisation–indole *N*-arylation†

**DOI:** 10.1039/d3ra02658b

**Published:** 2023-05-26

**Authors:** Mia Lintott, Alexis Perry

**Affiliations:** a Biosciences, University of Exeter Stocker Road Exeter EX4 4QD UK A.Perry@exeter.ac.uk

## Abstract

A microwave-promoted, one-pot, three-component synthesis of *N*-arylindoles has been developed, utilising sequential Fischer indolisation then copper(i)-catalysed indole *N*-arylation. Novel arylation conditions were identified that use a simple and cheap catalyst/base system (Cu_2_O/K_3_PO_4_) in an environmentally benign solvent (ethanol), with no requirement for ligands, additives or exclusion of air or water, and microwave irradiation enabled significant acceleration of this commonly sluggish process. These conditions were designed to dovetail with Fischer indolisation, and the resulting one-pot, two-step sequence is rapid (total reaction time = 40 minutes), operationally straightforward, generally high yielding and it draws upon readily available hydrazine, ketone/aldehyde and aryl iodide building blocks. This process shows broad substrate tolerance and we have demonstrated its utility in the synthesis of 18 *N*-arylindoles bearing varied and useful functionality.

## Abstract 2

One-pot, microwave-promoted Fischer indolisation–indole *N*-arylation enables straightforward, rapid synthesis of structurally-diverse *N*-arylindoles from widely-available precursors, using a simple, air-stable catalyst in a common, sustainable solvent.

## Graphical abstract

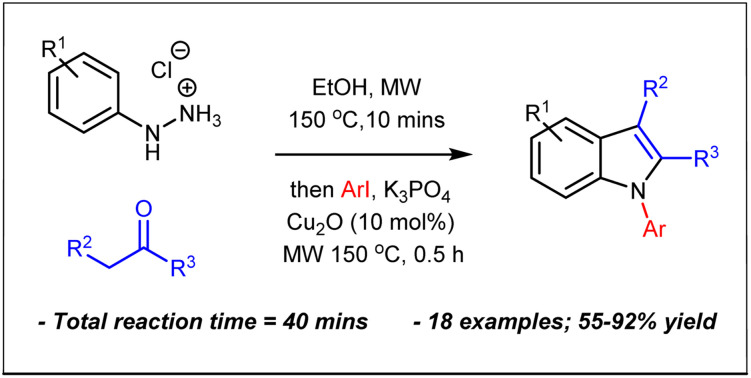

## Introduction

**Fig. 1:**
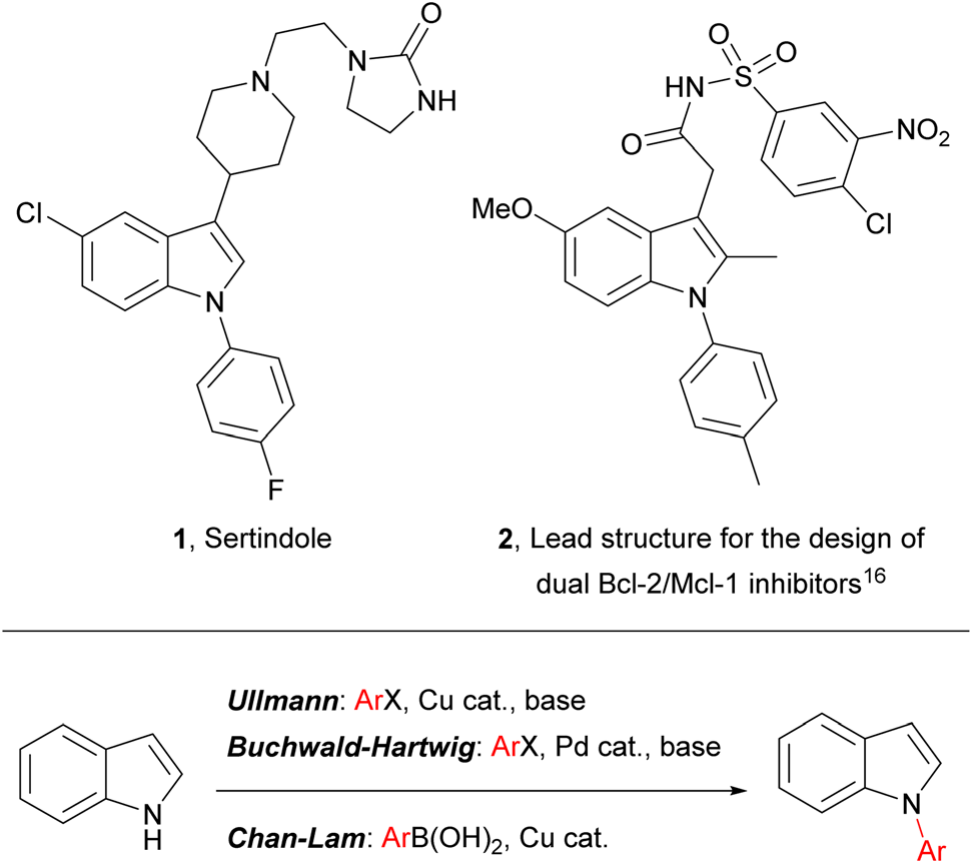
(Top) Representative examples of pharmaceutically significant *N*-aryl indoles; (bottom) common transition-metal-catalysed approaches to *N*-aryl indoles.

Indoles are widely encountered throughout nature and they are of prime importance in diverse areas of contemporary research, including pharmaceutical development (where they are considered to be a “privileged scaffold” for drug design),^1–4^ heterocyclic^5–9^ and asymmetric synthesis,^10,11^ and materials chemistry (*e.g.* in the development of composite materials for energy storage).^12–14^ Indoles bearing an aromatic substituent on the ring nitrogen – *N*-arylindoles – are an indole subset of biological and pharmaceutical significance, and *N*-arylindoles now account for several drug candidates and published drug discovery programmes (*e.g.*2), including one antipsychotic drug – sertindole (1) – on the market and embedded in current clinical practice (Fig. 1).^15–20^

Synthesis of *N*-aryl indoles is commonly achieved by metal-catalysed arylation of *N*-unsubstituted indoles, though non-metal-catalysed arylations, cyclisations of *N*-arylated indole precursors and reductive deoxygenation of *N*-arylated indolinones have also been documented and provide valuable complementary approaches.^21–23^ Arguably the most important metal-catalysed indole *N*-arylations are based upon either palladium or copper catalysts and include Ullmann condensation,^24,25^ Buchwald–Hartwig amination^26^ and Chan–Lam coupling (see Fig. 1).^27^ Between them, these complementary reactions provide relatively convenient and general access to a broad range of *N*-aryl indoles and, consequently, have been widely adopted. Recent developments have focussed on reducing the environmental footprint of *N*-arylation, by adapting methodology to tolerate environmentally benign solvents (*e.g.*, through use of micelles and phase-transfer catalysis)^28,29^ and lower temperatures (commonly, metal-catalysed indole *N*-arylations require heating >90 °C),^30^ and through development of C–H activation processes.^31,32^

**Fig. 2:**
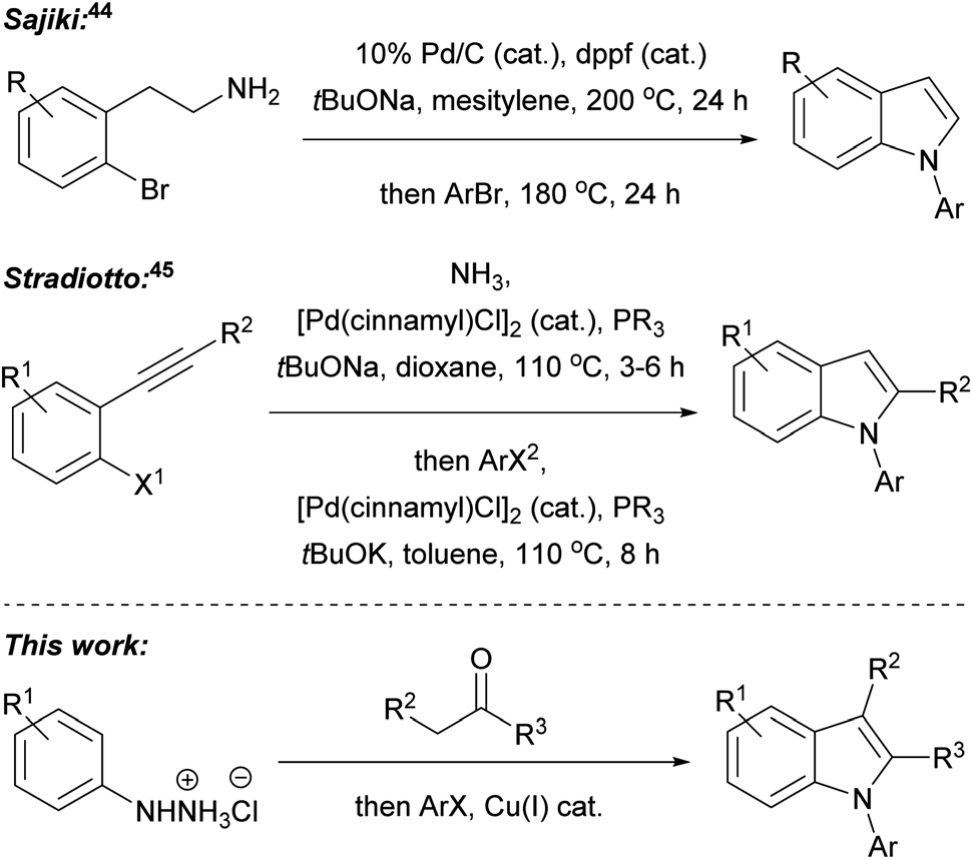
Published one-pot indole formation–*N*-arylation sequences, and the Fisher indolisation–*N*-arylation sequence described in this publication.

In our recent research, we developed a one-pot, two-step, three component Fischer indolisation–indole *N*-alkylation sequence for fast, convenient synthesis of *N*-alkylindoles.^33^ Subsequently, we have adapted this methodology for access to *N*-arylindoles, through the development of a highly efficient Fischer indolisation–indole *N*-arylation sequence, and this is the subject of this publication. One-pot, multi-step, multi-component reaction sequences can offer significant benefits over stepwise synthesis in terms of time, energy, yield, labour, consumables and cost, provided reactions with appropriately dovetailed characteristics can be identified.^34–36^ Fischer indolisation is a clean, high-yielding process which generates benign by-products and is effective across a range of solvents; consequently, this has been successfully employed in a variety of one-pot, multi-step reactions, often combining aryl hydrazone formation with Fischer indolisation (*e.g. via* addition of metalloimines to aryl hydrazines,^37^*via* addition of diazonium salts to acid chlorides^38^ or *via* Heck isomerisation).^39^ Indole *N*-arylation, on the other hand, is rather more complex, given the broad range of conditions, catalysts, ligands and substrates that it encompasses. We envisaged that although one set of arylation conditions chosen at random was unlikely to possess the latitude to dovetail with Fischer indolisation, somewhere in the swathe of available *N*-arylations, we would be able to identify appropriate conditions to promote effective one-pot indolisation–arylation. Various indole arylations have been used in one-pot reaction sequences, and these either involve sequential derivatisation of a pre-existing indole nucleus (*e.g.* through Cu(i)-promoted *N*-arylation then *C*-2 amidation;^40^ Suzuki arylation then Buchwald–Hartwig *N*-arylation;^41^ Ugi 4-component reaction then copper(i)-catalysed intramolecular *N*-arylation;^42^ and Pd(0) catalysed *N*-arylation then decarboxylative *C*-2 arylation^43^), or indole synthesis–*N*-arylation pathways (*e.g.* palladium-catalysed cyclisation of 2-bromophenethylamines then palladium-catalysed *N*-arylation;^44^ and indole synthesis *via* Pd(0)-catalysed cyclisation of aryl alkynes and ammonia, followed Pd(0)-catalysed *N*-arylation^45^), akin to the Fischer indolisation–*N*-arylation approach described herein (Fig. 2). Our overarching ideal was to develop methodology that would strike a balance between operational simplicity, practical and financial efficiency, and environmental impact. We considered that, amongst indole *N*-arylations, copper-catalysed processes were best placed to meet these criteria, whilst offering potential to pair with Fischer indolisation in a one-pot, multicomponent reaction sequence. The successful development of this chemistry is documented herein.

## Results and discussion

In developing a one-pot Fischer indolisation–indole *N*-alkylation sequence, we initially focussed on the two independent reactions before optimising conditions for the coupled process. Our investigations commenced with a brief exploration of environmentally acceptable solvents for Fischer indolisation, building on our previously established microwave-promoted Fischer indolisation–indole *N*-alkylation protocol.^33^ Using the reaction between phenylhydrazine hydrochloride (3) and butanone (4), we observed broad solvent tolerance for Fischer indolisation, with excellent yields of 2,3-dimethylindole (5) recorded in four trialled solvents (THF, 95%; 2-methyl THF, 96%; water, 92%; ethanol, 94%). This encouraging result gave us great flexibility in terms of solvent choice moving forward into copper-catalysed indole *N*-arylation, for which various solvents have been employed with success. From an environmental perspective, the use of water is clearly desirable, hence we focussed on conditions published by Yong *et al.*, in which copper-catalysed indole *N*-arylation proceeds under aqueous, basic conditions in the presence of a phase-transfer catalyst, albeit at elevated temperatures for an extended period.^29^ Using the reported conditions (Cu_2_O (10 mol%), TBAB (10 mol%), K_3_PO_4_ (2 eq.), water, 130 °C, sealed tube 24 h), we were able to access *N*-phenylindole 6a from 2,3-dimethylindole (5) and iodobenzene, although in only 23% yield. We suspect that this variance from considerably higher published yields is steric in origin; in contrast to our system, no example is presented incorporating substituents in the indole 2- or 3-positions. A marginal improvement – to 30% yield – was possible through use of microwave irradiation (300 W, 150 °C), simultaneously reducing the reaction time to 30 minutes. A more significant improvement was achieved by repeating these microwave-promoted conditions in an alternative sustainable solvent – ethanol – in which case we observed a 78% yield and no requirement for phase-transfer catalysis. With effective conditions and a common, sustainable solvent established for both indolisation and arylation, we performed the reactions as a one-pot sequence (conditions as described in Table 1, entry 1; an extra equivalent of base was included in the arylation step to neutralise the 1 equivalent of acid present from Fischer indolisation), and we were gratified to isolate 6a in 73% (85% per step). Further experimentation ascertained that yield decreased at a higher or lower arylation temperature (entries 2 and 3), and a shorter heating duration was also undesirable (entry 4). The reaction tolerated lower catalyst loading (61% with 5 mol% Cu_2_O) to a threshold around 2 mol% where yield fell away markedly, and higher catalyst loading had no impact on yield (entries 6–8); furthermore, it was observed that copper(i) iodide was a less effective arylation catalyst than Cu_2_O in this system (entry 9). Previous reports have documented a link between base strength and yield in related indole arylations,^46^ but in our case, use of a stronger base (sodium ethoxide) or a weaker base (caesium carbonate) resulted in reduced yields, and alternatives such as DBU were ineffective (entries 10–12). Reduction of base quantity, from 3 to 2 molar equivalents, also caused the reaction to fail (entry 13). Finally, whilst bromobenzene proved to be an ineffective coupling partner (entry 14), an increased quantity of iodobenzene, from 1.2 to 1.5 eq., enabled 6a to be synthesised in 80% (89% per step), and hence entry 15 represents our optimum conditions. It should be noted that use of 1.25 g mL^−1^ K_3_PO_4_ in ethanol results in a viscous, barely stirrable suspension; however, this does not seem to affect the success of the reaction and, moreover, doubling the volume of ethanol causes the yield to halve. This observation is consistent with previous studies in which higher reaction concentrations are correlated with yields in Ullmann couplings.^47^

**Table 1:** Optimisation of conditions for one-pot Fischer indolisation–*N*-arylation

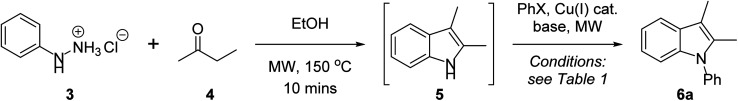					
Entry^a^	*T*/°C (time)	Base (eq.)	Catalyst (mol%)	PhX (eq.)	Yield^b^ (%)
1	150 (30 min)	K_3_PO_4_ (3 eq.)	Cu_2_O (10 mol%)	PhI (1.2 eq.)	73
2	130 (30 min)	K_3_PO_4_ (3 eq.)	Cu_2_O (10 mol%)	PhI (1.2 eq.)	12
3	170 (30 min)	K_3_PO_4_ (3 eq.)	Cu_2_O (10 mol%)	PhI (1.2 eq.)	48
4	150 (10 min)	K_3_PO_4_ (3 eq.)	Cu_2_O (10 mol%)	PhI (1.2 eq.)	44
5	150 (60 min)	K_3_PO_4_ (3 eq.)	Cu_2_O (10 mol%)	PhI (1.2 eq.)	69
6	150 (30 min)	K_3_PO_4_ (3 eq.)	Cu_2_O (20 mol%)	PhI (1.2 eq.)	73
7	150 (30 min)	K_3_PO_4_ (3 eq.)	Cu_2_O (5 mol%)	PhI (1.2 eq.)	61
8	150 (30 min)	K_3_PO_4_ (3 eq.)	Cu_2_O (2 mol%)	PhI (1.2 eq.)	8
9	150 (30 min)	K_3_PO_4_ (3 eq.)	CuI (10 mol%)	PhI (1.2 eq.)	51
10	150 (30 min)	NaOEt (3 eq.)	Cu_2_O (10 mol%)	PhI (1.2 eq.)	0
11	150 (30 min)	Cs_2_CO_3_ (3 eq.)	Cu_2_O (10 mol%)	PhI (1.2 eq.)	15
12	150 (30 min)	DBU (3 eq.)	Cu_2_O (10 mol%)	PhI (1.2 eq.)	0
13	150 (30 min)	K_3_PO_4_ (2 eq.)	Cu_2_O (10 mol%)	PhI (1.2 eq.)	13
14	150 (30 min)	K_3_PO_4_ (3 eq.)	Cu_2_O (10 mol%)	PhBr (1.2 eq.)	14
15	150 (30 min)	K_3_PO_4_ (3 eq.)	Cu_2_O (10 mol%)	PhI (1.5 eq.)	80

a All reaction sequences commenced with microwave-promoted Fischer indolisation of 3 (1.47 mmol) and 4 (1.54 mmol) in EtOH (0.75 mL), followed by addition of base, aryl halide and copper(i) catalyst and a further period of microwave heating.

b Isolated yield.

With optimised conditions in hand, we then explored the scope of this one-pot sequence. In general, we observed broad substrate tolerance in each of the three components (hydrazine, ketone/aldehyde, aryl iodide) and we were able access a breadth of *N*-arylated indole structures bearing a range of useful and varied functionality (Table 2). The reaction sequence could tolerate both electron-rich and electron-poor hydrazines and aryl iodides, including key components of pharmaceutically active structures, such as the 5-chloroindole and 4-fluorophenyl groups of sertindole (see Fig. 1), represented in 6c and 6s respectively. Similarly, the general tolerance towards ketone and aldehyde substrates encompassed 2-arylindoles (*e.g.*6l), which are under intense scrutiny for their anti-cancer,^48^ anti-inflammatory^49^ and fungicidal properties,^50^ and 2-carboxyindoles (*e.g.*6m), which have provided the core structure for the design of antidiabetic agents.^51,52^ We were pleased that this methodology allowed access to related heterocycles – including benzoindoles, tetrahydrocarbazoles, and *N*-thiophenyl- and *N*-pyridyl indoles (6b, 6k, 6n and 6o respectively) – as each heterocycle possesses its own unique chemical and biological profile, and, due to the inactivity of aryl bromides as substrates for *N*-arylation (see Table 1, entry 14), it was possible to synthesise brominated derivatives (*e.g.*6i), potentially giving rise to copper/palladium cross-coupling cascades.^53^ Indoles bearing bulky *N*-aryl substituents are inevitably prone to steric congestion and are absent from reports of related Ullmann arylations,^29^ hence it is notable that we were able to synthesise 6b, 6e and 6l, in which *N*-arylation may be impeded by substituents in indole 2 and/or 7 positions, albeit in reduced yields. Further exploration of steric influence demonstrated that yields of *N*-(2-, 3- and 4-chlorophenyl)indoles 6p, 6q and 6r increased as the chlorine atom was placed further from the indole nucleus. Beyond limitations imposed by steric effects, indoles 6f, 6j and 6v were inaccessible using this methodology. Highly electron-poor hydrazones are documented as unwilling substrates in Fischer indolisation;^54,55^ accordingly, 4-nitrophenylhydrazine was an unsuccessful substrate in this reaction sequence (hence 6f was not synthesised). Use of 4-nitroiodobenzene as an arylation substrate resulted in the production of a sticky, black tar with no evidence to suggest formation of 6v, and it is likely that we were unable to access ester 6j due to its susceptibility to base-promoted thermal decarboxylation – this is a documented synthetic transformation and, correspondingly, we were able to identify decarboxylated 2-methyl-1-phenyl indole within the crude reaction product.^56^

**Table 2:** Scope of one-pot Fischer indolisation–indole *N*-arylation. Reactions were conducted using conditions defined in Table 1, entry 15, using varied hydrazine hydrochloride (1.47 mmol), ketone/aldehyde (1.54 mmol, 1.05 eq.) and aryl iodide (2.21 mmol, 1.5 eq.) components

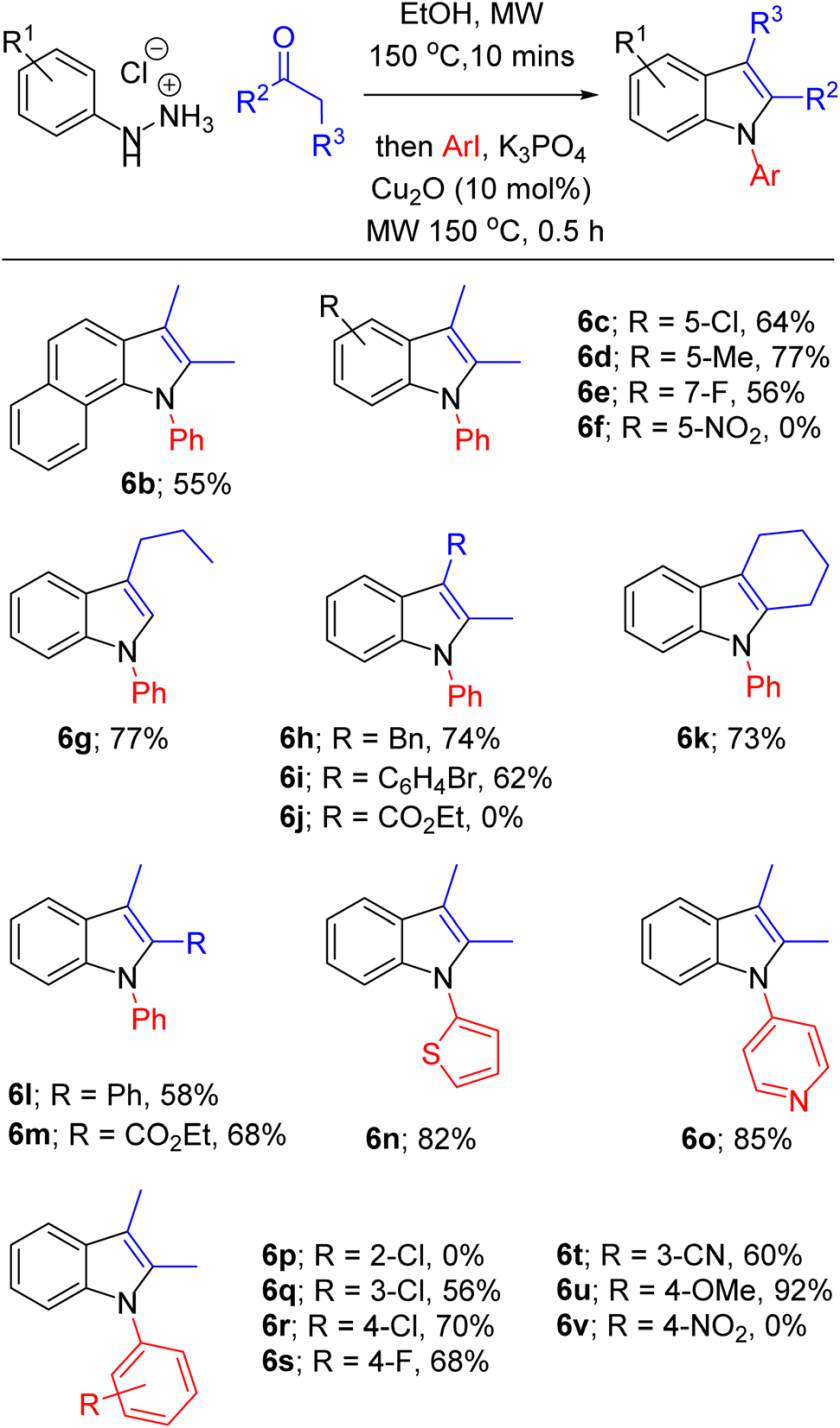

## Conclusion

In summary, we have developed one-pot, three-component Fischer indolisation–indole *N*-arylation as a convenient, efficient sequence for the synthesis of valuable *N*-arylindole products. The reaction cascade uses a sustainable solvent, ethanol, in combination with inexpensive and easily handled reagents and catalysts, and employs microwave irradiation of both indolisation and arylation steps to reduce time and energy usage. Its broad substrate tolerance has enabled synthesis of a range of interesting arylated indole structures possessing useful functionality, in good overall yields. We have achieved this through the development of bespoke conditions for copper-catalysed indole *N*-arylation that are designed to dovetail with conditions for Fischer indolisation. Although conceived within a multicomponent reaction regimen, it is worth noting that as stand-alone *N*-arylation methodology, these conditions complement and improve upon published copper-catalysed arylation protocols, in that there is no requirement for ligands or additives, exclusion of air or water is not required, the reaction proceeds rapidly in an environmentally benign solvent and shows broad substrate tolerance. Overall, this research demonstrates that one-pot, multi-step, microwave-promoted synthesis is a powerful and effective strategy in the development of convenient and user-friendly methodologies for the synthesis of important molecules.

## Conflicts of interest

There are no conflicts to declare.

## Supplementary Material

RA-013-D3RA02658B-s001
