# Supplementary material for: Straightforward synthesis of N-arylindoles via one-pot Fischer indolisation–indole N-arylation
Source: RSC Adv. 2023 May 26;13(23):15993–7. doi: 10.1039/d3ra02658b (PMC10214496; doi:10.1039/d3ra02658b)
Supplement: RA-013-D3RA02658B-s001 [file RA-013-D3RA02658B-s001.pdf]

Electronic Supplementary Material (ESI) for RSC Advances.
This journal is © The Royal Society of Chemistry 2023

Supporting Information for:

Straightforward synthesis of N-arylindoles via one-pot Fischer indolisation–indole N-arylation

Mia Lintott[a] and Alexis Perry[a]*

a **– Biosciences, University of Exeter, Stocker Road, Exeter EX4 4QD, UK**

*** – A.Perry@exeter.ac.uk**

Contents

**1.**	**General considerations**	**1**
**2.**	**Experimental methods and characterisation data for *N*-arylindoles 6a-6u**	**2**
**3.**	**^{1}H and ^{13}C NMR spectra for *N*-arylindoles 6a-6u**	**10**
**4.**	**References for supporting information**	**28**

1. General considerations

Solvents and reagents were used as commercially supplied. Water refers to deionised water. Analytical thin layer chromatography was carried out using Merck Kieselgel 60 F254, coated on aluminium plates, with visualisation of spots where necessary by quenching of UV(254 nm) fluorescence. Silica gel with particle size 40–63 µm was used for flash chromatography. Microwave reactions were performed in a CEM Discover microwave in 10 mL, thick-walled microwave tubes, sealed with septum caps and were magnetically stirred. Infrared spectra were recorded as thin films using attenuated total reflectance with a Nicolet iS5 FTIR spectrometer. Mass spectra were recorded on a QToF 6520 mass spectrometer (Agilent Technologies, Palo Alto, USA). ^{1}H NMR, ^{13}C NMR and ^{19}F NMR spectra were recorded at 400 MHz, 100 MHz and 376 MHz respectively, using a Bruker Avance III HD400 spectrometer. Chemical shifts are quoted in ppm relative to tetramethylsilane for ^{1}H NMR and ^{13}C NMR, the residual solvent peak being used for referencing purposes, and relative to trichlorofluoromethane for ^{19}F NMR. Coupling constants are quoted to the nearest 0.1 Hz with peak multiplicities for single resonances being labelled as: s, singlet; d, doublet; t, triplet; q, quartet; m, unresolved multiplet.

2. Experimental methods and characterisation data for *N*-arylindoles 6a-6u

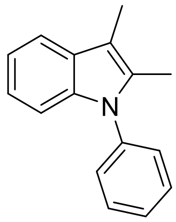

General Procedure, illustrated by the synthesis of 2,3-dimethyl-1-phenylindole[1] 6a

Butanone (138 µL, 1.54 mmol, 1.05 eq.), phenylhydrazine hydrochloride (213 mg, 1.47 mmol, 1 eq.) and ethanol (750 µL) were combined in a thick-walled microwave tube. The tube was capped with a septum and heated to 150 °C using microwave irradiation (maximum power = 300 W; time to reach 150 °C = 3.5 minutes), then held at this temperature for 10 minutes. After cooling to room temperature, iodobenzene (246 µL, 2.21 mmol, 1.5 eq.), tripotassium phosphate (936 mg, 4.41 mmol, 3 eq.) then copper(I) oxide (21 mg, 0.147 mmol, 0.1 eq.) were added to the stirred reaction mixture, which was then recapped and heated to 150 °C using microwave irradiation (maximum power = 300 W; time to reach 150 °C = 3 minutes), then held at this temperature for 30 minutes. After cooling to room temperature, the reaction mixture was diluted with ethanol (5 mL) and filtered through Celite®, eluting with ethanol, then concentrated under reduced pressure. The crude indole was purified by flash chromatography, eluting with 2% ethyl acetate in hexane, to give 2,3-dimethyl-1-phenylindole **6a** (260 mg, 80%), spectroscopically identical to that previously reported.[1]

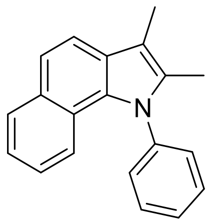

2,3-Dimethyl-1-phenylbenzo[g]indole 6b

By the same general procedure, butanone (138 µL, 1.54 mmol, 1.05 eq.), 1-naphthylhydrazine hydrochloride (286 mg, 1.47 mmol, 1 eq.) and iodobenzene (246 µL, 2.21 mmol, 1.5 eq.) gave indole **6b** (221 mg, 55%) as a yellow oil, R_f 0.3 (hexane); ν_{max} = 3054, 2915, 2858, 1595, 1522, 1496, 1406, 1386, 1274, 1009, 803, 744, 712 and 697 cm^{-1}; δH(400 MHz, $CDCl_3$) 7.78 (1 H, d, *J* 8.1, 6), 7.60 (1 H, d, *J* 8.6, 4), 7.53-7.46 (3 H, m, 3′, 4′ and 5′), 7.44 (1 H, d, *J* 8.6, 5), 7.30 (2 H, d, *J* 8.1, 2′ and 6′), 7.19-7.13 (1 H, m, 7), 7.02-6.96 (2 H, m, 8 and 9), 2.30 (3 H, s, 3-Me) and 2.06 (3 H, s, 2-Me); δC(100 MHz, $CDCl_3$) 141.1 (1′), 132.7 (2), 131.0 (5a), 130.2 (9b), 129.8 (3′ and 5′), 129.3 (2′ and 6′), 129.0 (6), 128.7 (4′), 125.3 (3a), 124.6 (8), 122.7 (7), 122.3 (9a), 120.6 (5), 120.5 (9), 118.4 (4), 108.8 (3), 10.9 (2-Me) and 9.0 (3-Me); HRMS-ES (*m/z*): Found: 272.1429 (MH^+, $C_{20}H_{18}N$ requires: 272.1434).

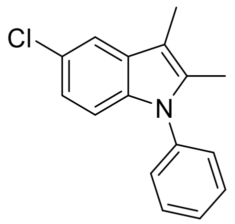

5-Chloro-2,3-dimethyl-1-phenylindole 6c

By the same general procedure, butanone (138 µL, 1.54 mmol, 1.05 eq.), 4-chlorophenylhydrazine hydrochloride (263 mg, 1.47 mmol, 1 eq.) and iodobenzene (246 µL, 2.21 mmol, 1.5 eq.) gave indole **6c** (242 mg, 64%) as an orange oil, R_f 0.3 (hexane); ν_{max} = 2917, 2858, 1596, 1499, 1460, 1395, 1372, 1267, 1075, 939, 917, 859, 793, 761 and 702 cm^{-1}; δH(400 MHz, $CDCl_3$) 7.39-7.32 (3 H, m, 4, 3′ and 5′), 7.28 (1 H, t, *J* 8.1, 4′), 7.13 (2 H, d, *J* 8.1, 2′ and 6′), 6.88 (1 H, dd, *J* 8.5 and 1.8, 6), 6.84 (1 H, d, *J* 8.5, 7), 2.14 (3 H, s, 3-Me) and 2.07 (3 H, s, 2-Me); δC(100 MHz, $CDCl_3$) 138.0 (1′), 135.8 (7a), 134.5 (2), 130.0 (3a), 129.6 (3′ and 5′), 128.0 (2′ and 6′), 127.8 (4′), 125.2 (5), 121.3 (6), 117.5 (4), 110.8 (7), 107.8 (3), 11.1 (2-Me) and 8.9 (3-Me); HRMS-ES (*m/z*): Found: 256.0846 (MH^+, $C_{16}H_{15}{}^{35}ClN$ requires: 256.0888).

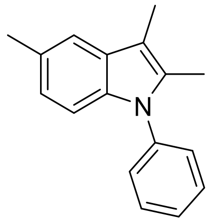

2,3,5-Trimethyl-1-phenylindole[2] 6d

By the same general procedure, butanone (138 µL, 1.54 mmol, 1.05 eq.), 4-methylphenylhydrazine hydrochloride (233 mg, 1.47 mmol, 1 eq.) and iodobenzene (246 µL, 2.21 mmol, 1.5 eq.) gave indole **6d** (265 mg, 77%), spectroscopically identical to that previously reported.[2]

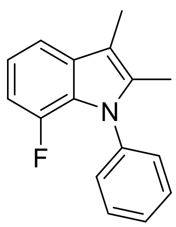

7-Fluoro-2,3-dimethyl-1-phenylindole 6e

By the same general procedure, butanone (138 µL, 1.54 mmol, 1.05 eq.), 2-fluorophenylhydrazine hydrochloride (239 mg, 1.47 mmol, 1 eq.) and iodobenzene (246 µL, 2.21 mmol, 1.5 eq.) gave indole **6e** (198 mg, 56%) as an orange oil, R_f 0.3 (hexane); ν_{max} = 3063, 2917, 1597, 1572, 1499, 1450, 1275, 1240, 1210, 1052, 776, 725 and 696 cm^{-1}; δH(400 MHz, $CDCl_3$) 7.41-7.32 (3 H, m, 3′, 4′ and 5′), 7.23 (2 H, d, *J* 8.1, 2′ and 6′), 7.22 (1 H, d, *J* 7.9, 4), 6.92 (1 H, dt, *J* 7.9 and 4.4, 5), 6.69 (1 H, dd, *J* 12.3 and 7.9, 6), 2.21 (3 H, s, 3-Me) and 2.06 (3 H, s, 2-Me); δC(100 MHz, $CDCl_3$) 149.3 (d, *J* 244, 7), 139.3 (1′), 134.6 (2), 132.8 (d, *J* 5, 3a), 128.7 (3′ and 5′), 128.3 (2′ and 6′), 127.8 (4′), 124.6 (d, *J* 8, 7a), 119.3 (d, *J*

7, 5), 113.7 (d, *J* 3, 4), 108.7 (3), 107.1 (d, *J* 18, 6), 10.8 (2-Me) and 9.1 (3-Me); δF (376 MHz, $CDCl_3$) –131.8; HRMS-ES (*m/z*): Found: 240.1162 (MH^+, $C_{16}H_{15}FN$ requires: 240.1183).

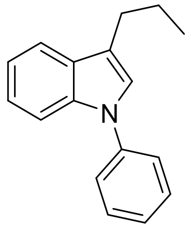

Phenyl-3-propylindole[3] 6g

By the same general procedure, valeraldehyde (164 µL, 1.54 mmol, 1.05 eq.), phenylhydrazine hydrochloride (213 mg, 1.47 mmol, 1 eq.) and iodobenzene (246 µL, 2.21 mmol, 1.5 eq.) gave indole **6g** (267 mg, 77%), spectroscopically identical to that previously reported.[3]

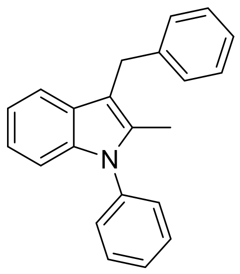

3-Benzyl-2-methyl-1-phenylindole 6h

By the same general procedure, benzylacetone (231 µL, 1.54 mmol, 1.05 eq.), phenylhydrazine hydrochloride (213 mg, 1.47 mmol, 1 eq.) and iodobenzene (246 µL, 2.21 mmol, 1.5 eq.) gave indole **6h** (324 mg, 74%) as an orange oil, R_f 0.3 (3% EtOAc in hexane); ν_{max} = 3025, 2915, 1596, 1568, 1499, 1458, 1399, 1321, 1073, 1016, 916, 739 and 699 cm^{-1}; δH(400 MHz, $CDCl_3$) 7.42-7.35 (3 H, m, 4, 3' and 5'), 7.30 (1 H, t, *J* 7.5, 4'), 7.23 (2 H, d, *J* 7.5, 2' and 6'), 7.17 (2 H, d, *J* 7.4, Bn 2 and Bn 6), 7.14 (2 H, t, *J* 7.4, Bn 3 and Bn 5), 7.05 (1 H, t, *J* 7.4, Bn 4), 7.03-6.94 (3 H, m, 5, 6 and 7), 4.05 (2 H, s, CH_2) and 2.14 (3 H, s, 2-Me); δC(100 MHz, $CDCl_3$) 141.7 (Bn 1), 138.3 (1'), 137.6 (7a), 133.9 (2), 129.5 (3' and 5'), 129.4 (3a), 128.4 (Bn 3 and Bn 5), 128.4 (Bn 2 and Bn 6), 128.2 (2' and 6'), 127.7 (4'), 125.8 (Bn 4), 121.3 (6), 119.8 (5), 118.4 (4), 111.5 (3), 110.0 (7), 30.6 (CH_2) and 11.3 (2-Me); HRMS-ES (*m/z*): Found: 298.1584 (MH^+, $C_{22}H_{20}N$ requires: 298.1590).

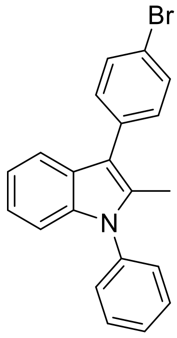

3-(4-Bromophenyl)-2-methyl-1-phenylindole 6i

By the same general procedure, 4-bromophenylacetone (231 µL, 1.54 mmol, 1.05 eq.), phenylhydrazine hydrochloride (213 mg, 1.47 mmol, 1 eq.) and iodobenzene (246 µL, 2.21 mmol, 1.5 eq.) gave indole **6i** (330 mg, 62%) as an amorphous yellow solid, R_f 0.3 (hexane); ν_{max} = 3028, 2920, 1594, 1543, 1496, 1486, 1456, 1171, 1072, 1007, 924, 822, 742, 698 and 656 cm^{-1}; δH(400 MHz, $CDCl_3$) 7.62-7.50 (1 H, m, 4), 7.53 (2 H, d, *J* 8.4, 2'' and 6''), 7.49 (2 H, t, *J* 7.4, 3' and 5'), 7.41 (1 H, t, *J* 7.4, 4'), 7.36 (2 H, d, *J* 8.4, 3'' and 5''), 7.33 (2 H, d, *J* 7.4, 2' and 6'), 7.14-7.02 (3 H, m, 5, 6 and 7) and 2.25 (3 H, s, 2-Me); δC(100 MHz, $CDCl_3$) 137.7 (1'), 137.6 (7a), 134.5 (4''), 133.7 (2), 131.7 (2'' and 6''), 131.2 (3'' and 5''), 129.6 (3' and 5'), 129.2 (3a), 128.2 (2' and 6'), 128.0 (4'), 121.9 (6), 120.6 (5), 119.7 (1''), 118.4 (4), 114.2 (3), 110.2 (7) and 12.0 (2-Me); HRMS-ES (*m/z*): Found: 362.0524 (MH^+, $C_{21}H_{17}{}^{79}BrN$ requires: 362.0539).

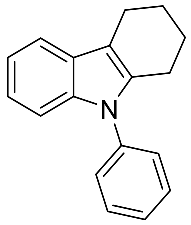

9-Phenyl-2,3,4,9-tetrahydrocarbazole[4] 6k

By the same general procedure, cyclohexanone (159 µL, 1.54 mmol, 1.05 eq.), phenylhydrazine hydrochloride (213 mg, 1.47 mmol, 1 eq.) and iodobenzene (246 µL, 2.21 mmol, 1.5 eq.) gave indole **6k** (264 mg, 73%), spectroscopically identical to that previously reported.[4]

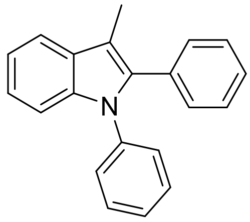

3-Methyl-1,2-diphenylindole[5] 6l

By the same general procedure, propiophenone (205 µL, 1.54 mmol, 1.05 eq.), phenylhydrazine hydrochloride (213 mg, 1.47 mmol, 1 eq.) and iodobenzene (246 µL, 2.21 mmol, 1.5 eq.) gave indole **6l** (242 mg, 58%), spectroscopically identical to that previously reported.[5]

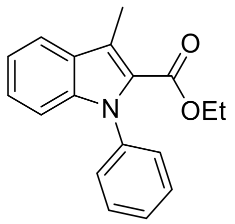

Ethyl 3-methyl-1-phenylindole-2-carboxylate[6] 6m

By the same general procedure, ethyl-2-oxobutyrate (203 µL, 1.54 mmol, 1.05 eq.), phenylhydrazine hydrochloride (213 mg, 1.47 mmol, 1 eq.) and iodobenzene (246 µL, 2.21 mmol, 1.5 eq.) gave indole **6m** (279 mg, 68%), spectroscopically identical to that previously reported.[6]

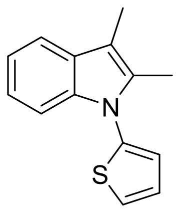

2,3-Dimethyl-1-(thiophen-2-yl)indole 6n

By the same general procedure, butanone (138 µL, 1.54 mmol, 1.05 eq.), phenylhydrazine hydrochloride (213 mg, 1.47 mmol, 1 eq.) and 2-iodothiophene (244 µL, 2.21 mmol, 1.5 eq.) gave indole **6n** (274 mg, 82%) as a brown oil, R_f 0.3 (hexane); ν_{max} = 3027, 2914, 2858, 1548, 1457, 1441, 1325, 1201, 1129, 1014, 896, 842, 738 and 694 cm^{-1}; δH(400 MHz, $CDCl_3$) 7.44-7.39 (1 H, m, 4), 7.19 (1 H, dd, *J* 5.6 and 1.2, 5′), 7.12-7.01 (3 H, m, 5, 6 and 7), 6.97 (1 H, dd, *J* 5.6 and 3.6, 4′), 6.89 (1 H, dd, *J* 3.6 and 1.2, 3′), 2.20 (3 H, s, 3-Me) and 2.16 (3 H, s, 2-Me); δC(100 MHz, $CDCl_3$) 139.8 (2′), 138.7 (7a), 133.9 (2), 129.1 (3a), 125.9 (4′), 125.4 (3′), 124.5 (5′), 121.7 (6), 120.1 (5), 117.9 (4), 110.1 (7), 108.8 (3), 10.7 (2-Me) and 9.0 (3-Me); HRMS-ES (*m/z*): Found: 228.0833 (MH^+, $C_{14}H_{14}NS$ requires: 228.0842).

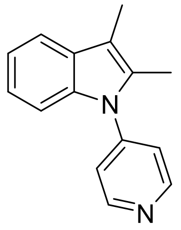

2,3-Dimethyl-1-(pyridin-4-yl)indole 6o

By the same general procedure, butanone (138 µL, 1.54 mmol, 1.05 eq.), phenylhydrazine hydrochloride (213 mg, 1.47 mmol, 1 eq.) and 4-iodopyridine (453 mg, 2.21 mmol, 1.5 eq.) gave indole **6o** (278 mg, 85%) as an amorphous orange solid, R_f 0.2 (25% EtOAc in hexane); ν_{max} = 3026, 2905, 2849, 1589, 1558, 1502, 1417, 1155, 1135, 991, 887, 846, 830, 743 and 636 cm^{-1}; δH(400 MHz, $CDCl_3$) 8.59 (2 H, d, *J* 3.9, 2′ and 6′), 7.41 (1 H, d, *J* 7.1, 4), 7.15-7.09 (3 H, m, 7, 3′ and 5′), 7.03 (1 H, t, *J* 7.1, 5), 7.02 (1 H, t, *J* 7.1, 6), 2.18 (3 H, s, 3-Me) and 2.16 (3 H, s, 2-Me); δC(100 MHz, $CDCl_3$) 151.2 (2′ and 6′), 145.9 (4′), 136.2 (7a), 131.7 (2), 129.6 (3a), 122.1 (6), 121.8 (3′ and 5′), 120.6 (5), 118.4

(4), 110.5 (3), 109.6 (7), 11.3 (2-Me) and 8.9 (3-Me); HRMS-ES (*m/z*): Found: 223.1238 (MH^+, $C_{15}H_{15}N_2$ requires: 223.1230).

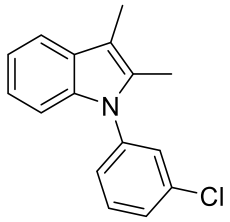

1-(3-Chlorophenyl)-2,3-dimethylindole 6q

By the same general procedure, butanone (138 µL, 1.54 mmol, 1.05 eq.), phenylhydrazine hydrochloride (213 mg, 1.47 mmol, 1 eq.) and 1-chloro-3-iodobenzene (274 µL, 2.21 mmol, 1.5 eq.) gave indole **6q** (211 mg, 56%) as a yellow oil, R_f 0.3 (hexane); ν_{max} = 3054, 2915, 2858, 1590, 1572, 1477, 1234, 1151, 1092, 888, 783, 737 and 693 cm^{-1}; δH(400 MHz, $CDCl_3$) 7.45 (1 H, d, *J* 8.3, 4), 7.39 (1 H, d, *J* 8.0, 5′), 7.32 (1 H, dt, *J* 8.0 and 1.7, 6′), 7.27 (1 H, t, *J* 1.7, 2′), 7.15 (1 H, dt, *J* 8.0 and 1.7, 4′), 7.10-7.01 (3 H, m, 5,6 and 7), 2.23 (3 H, s, 3-Me) and 2.16 (3 H, s, 2-Me); δC(100 MHz, $CDCl_3$) 139.8 (1′), 137.3 (7a), 135.0 (3′), 132.6 (2), 130.5 (5′), 129.2 (3a), 128.3 (2′), 127.7 (6′), 126.3 (4′), 121.7 (6), 120.1 (5), 118.2 (4), 109.7 (7), 108.9 (3), 11.1 (2-Me) and 9.1 (3-Me); HRMS-ES (*m/z*): Found: 256.0830 (MH^+, $C_{16}H_{15}{}^{35}ClN$ requires: 256.0888).

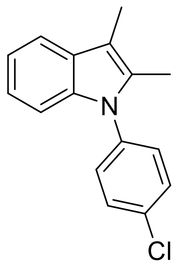

1-(4-Chlorophenyl)-2,3-dimethylindole 6r

By the same general procedure, butanone (138 µL, 1.54 mmol, 1.05 eq.), phenylhydrazine hydrochloride (213 mg, 1.47 mmol, 1 eq.) and 1-chloro-4-iodobenzene (527 mg, 2.21 mmol, 1.5 eq.) gave indole **6r** (263 mg, 70%) as an amorphous yellow solid, R_f 0.3 (hexane); ν_{max} = 3020, 2951, 2910, 1593, 1491, 1458, 1133, 1088, 1012, 934, 886, 739 and 718 cm^{-1}; δH(400 MHz, $CDCl_3$) 7.42 (1 H, d, *J* 7.0, 4), 7.34 (2 H, d, *J* 8.5, 3′ and 5′), 7.12 (2 H, d, *J* 8.5, 2′ and 6′), 7.05-6.93 (3 H, m, 5, 6 and 7), 2.20 (3 H, s, 3-Me) and 2.10 (3 H, s, 2-Me); δC(100 MHz, $CDCl_3$) 137.2 (7a), 137.0 (1′), 133.2 (4′), 132.6 (2), 129.7 (3′ and 5′), 129.3 (2′ and 6′), 129.0 (3a), 121.5 (6), 119.8 (5), 118.1 (4), 109.6 (7), 108.6 (3), 11.0 (2-Me) and 9.0 (3-Me); HRMS-ES (*m/z*): Found: 256.0785 (MH^+, $C_{16}H_{15}{}^{35}ClN$ requires: 256.0888).

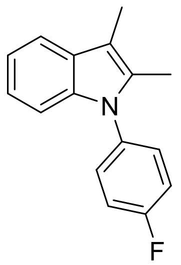

1-(4-Fluorophenyl)-2,3-dimethylindole 6s

By the same general procedure, butanone (138 µL, 1.54 mmol, 1.05 eq.), phenylhydrazine hydrochloride (213 mg, 1.47 mmol, 1 eq.) and 4-fluoroiodobenzene (255 µL, 2.21 mmol, 1.5 eq.) gave indole **6s** (239 mg, 68%) as a yellow oil, R_f 0.3 (hexane); ν_{max} = 3062, 2917, 1590, 1491, 1450, 1268, 1238, 1199, 1052, 776, 720 and 696 cm^{-1}; δH(400 MHz, $CDCl_3$) 7.75 (1 H, d, *J* 7.5, 4), 7.42 (2 H, dd, *J* 8.7 and 5.0, 2′ and 6′), 7.34 (2 H, t, *J* 8.7, 3′ and 5′), 7.33 (1 H, t, *J* 7.5, 5), 7.31 (1 H, t, *J* 7.5, 6), 7.25 (1 H, d, *J* 7.5, 7), 2.53 (3 H, s, 3-Me) and 2.39 (3 H, s, 2-Me); δC(100 MHz, $CDCl_3$) 160.5 (d, *J* 246, 4′), 136.3 (7a), 133.2 (d, *J* 3, 1′), 131.6 (2), 128.6 (d, *J* 9, 2′ and 6′), 127.7 (3a), 120.2 (6), 118.5 (5), 116.8 (4), 115.1 (d, *J* 23, 3′ and 5′), 108.4 (7), 106.9 (3), 9.7 (3-Me) and 7.7 (2-Me); δF (376 MHz, $CDCl_3$) –114.4; HRMS-ES (*m/z*): Found: 240.1165 (MH^+, $C_{16}H_{15}FN$ requires: 240.1183).

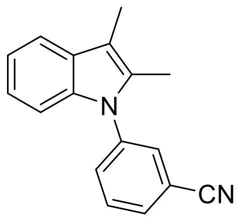

3-(2,3-Dimethylindol-1-yl)benzonitrile 6t

By the same general procedure, butanone (138 µL, 1.54 mmol, 1.05 eq.), phenylhydrazine hydrochloride (213 mg, 1.47 mmol, 1 eq.) and 3-iodobenzonitrile (506 mg, 2.21 mmol, 1.5 eq.) gave indole **6t** (217 mg, 60%) as an amorphous orange solid, R_f 0.3 (10% EtOAc in hexane); ν_{max} = 3018, 2938, 2908, 2232, 1599, 1577, 1462, 1436, 1313, 1171, 1094, 1016, 906, 800, 742 and 694 cm^{-1}; δH(400 MHz, $CDCl_3$) 7.60 (1 H, dt, *J* 7.4 and 1.5, 4′), 7.57-7.50 (2 H, m, 2′ and 5′), 7.49 (1 H, dt, *J* 7.9 and 1.5, 6′), 7.44 (1 H, dd, *J* 7.0 and 1.3, 4), 7.06 (1 H, td, *J* 7.0 and 1.3, 5), 7.03 (1 H, td, *J* 7.0 and 1.3, 6), 6.97 (1 H, dd, *J* 7.0 and 1.3, 7), 2.22 (3 H, s, 3-Me) and 2.14 (3 H, s, 2-Me); δC(100 MHz, $CDCl_3$) 138.3 (1′), 135.9 (7a), 131.4 (6′), 131.0 (2), 130.1 (2′), 129.7 (5′), 129.4 (4′), 128.1 (3a), 120.8 (6), 119.2 (5), 117.2 (4), 116.9 (CN), 112.6 (3′), 108.4 (3), 108.1 (7), 9.9 (2-Me) and 7.8 (3-Me); HRMS-ES (*m/z*): Found: 247.1224 (MH^+, $C_{17}H_{15}N_2$ requires: 247.1230).

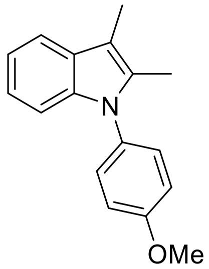

1-(4-Methoxyphenyl)-2,3-dimethylindole[7] 6u

By the same general procedure, butanone (138 µL, 1.54 mmol, 1.05 eq.), phenylhydrazine hydrochloride (213 mg, 1.47 mmol, 1 eq.) and 4-iodoanisole (517 mg, 2.21 mmol, 1.5 eq.) gave indole **6u** (341 mg, 92%) as a colourless oil, spectroscopically identical to that previously reported.[7]

3. ^{1}H and ^{13}C spectra for 6a-6u

^{1}H and ^{13}C spectra for **6a**

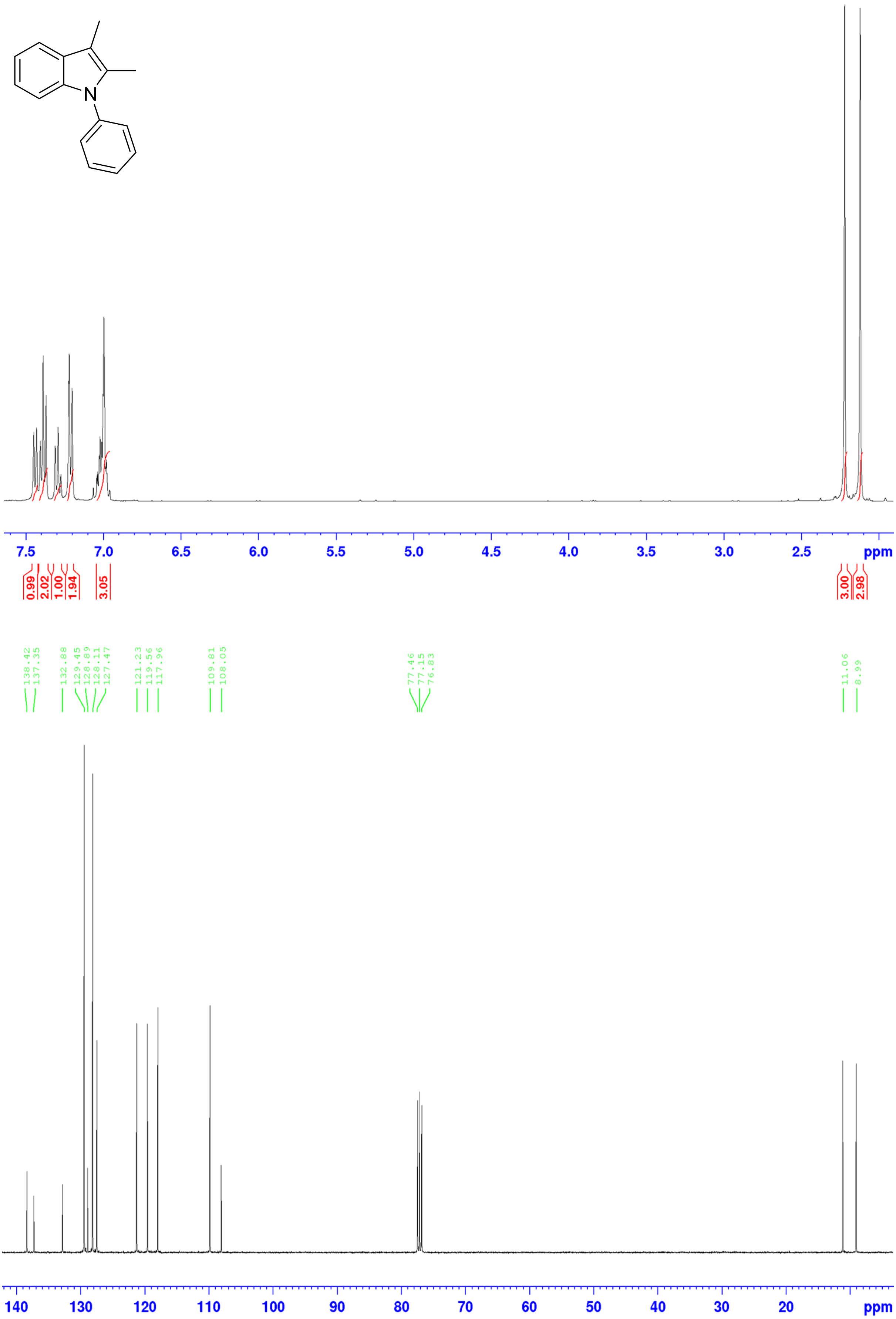

^{1}H and ^{13}C spectra for **6b**

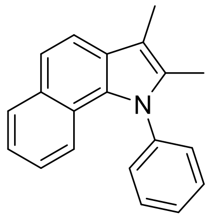

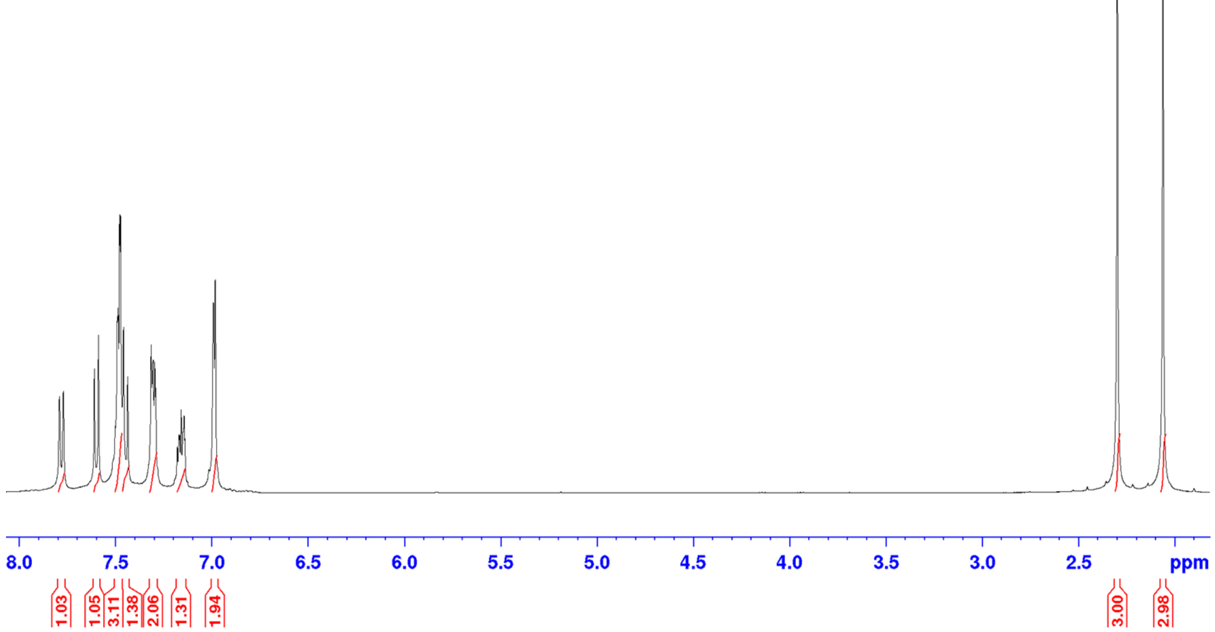

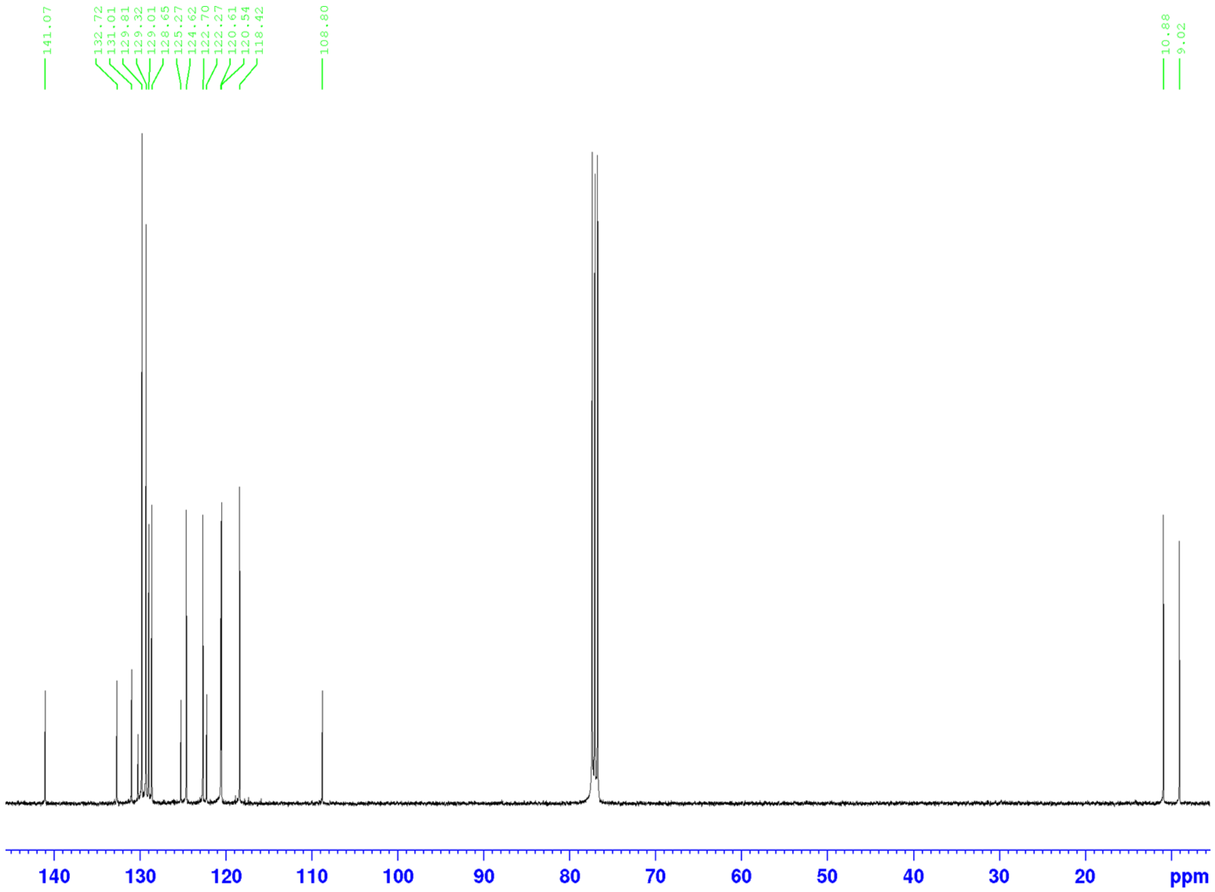

^{1}H and ^{13}C spectra for **6c**

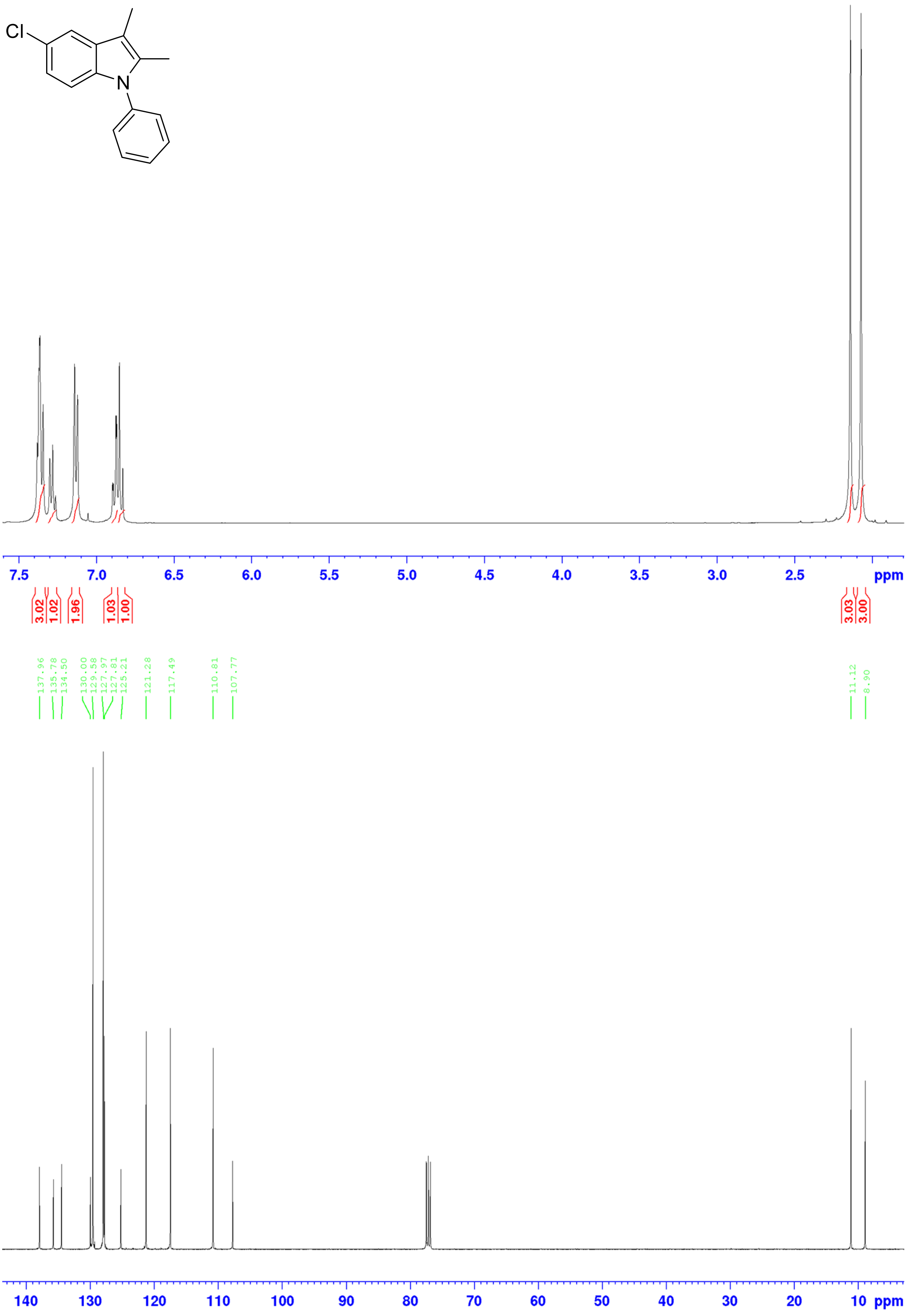

^{1}H and ^{13}C spectra for **6d**

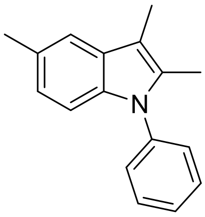

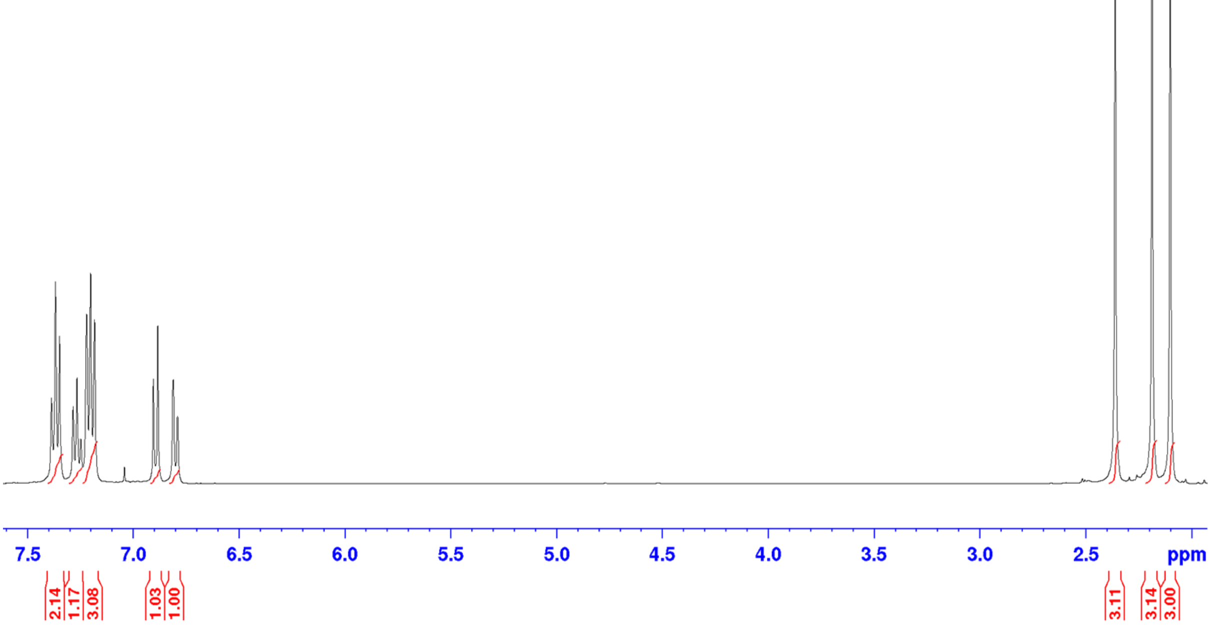

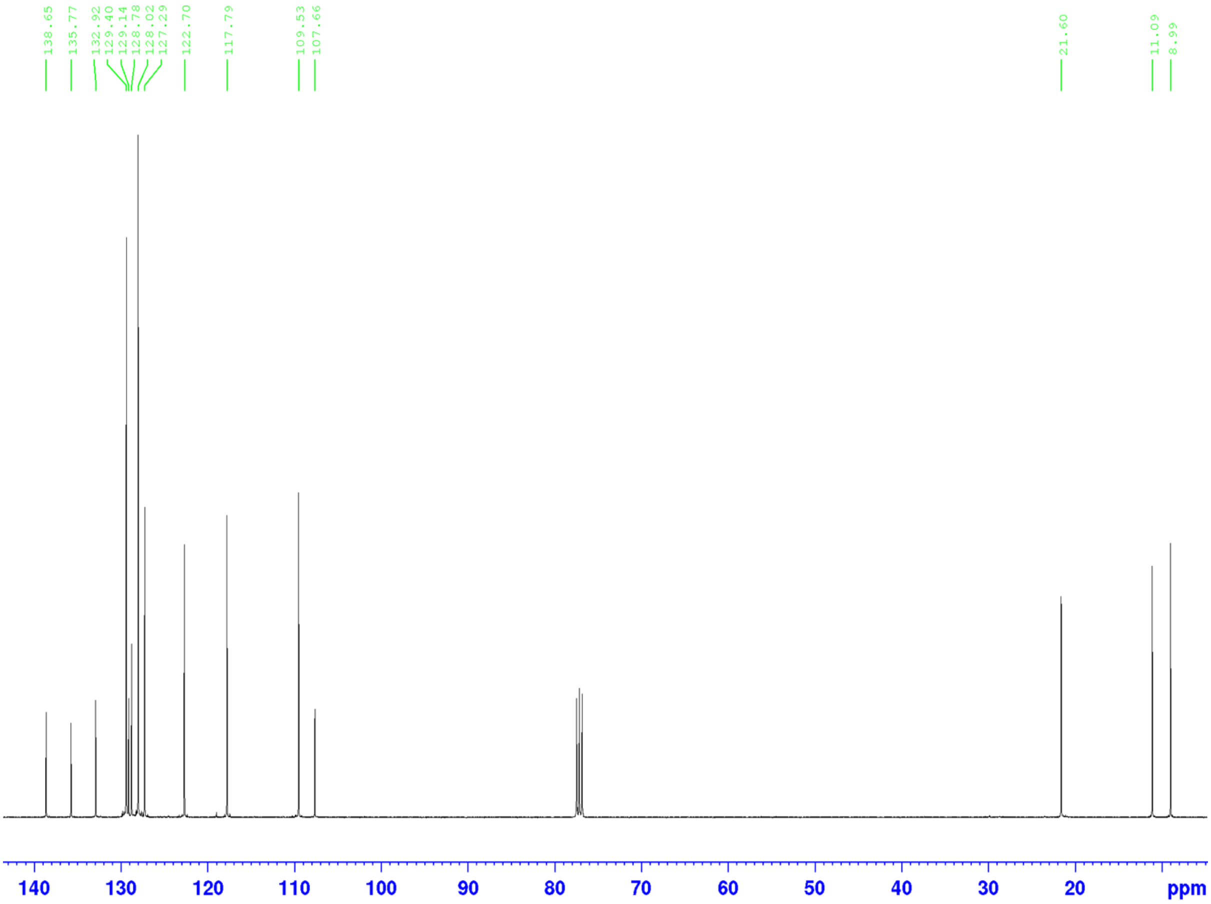

^{1}H and ^{13}C spectra for **6e**

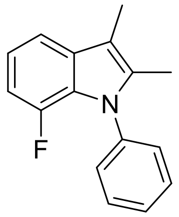

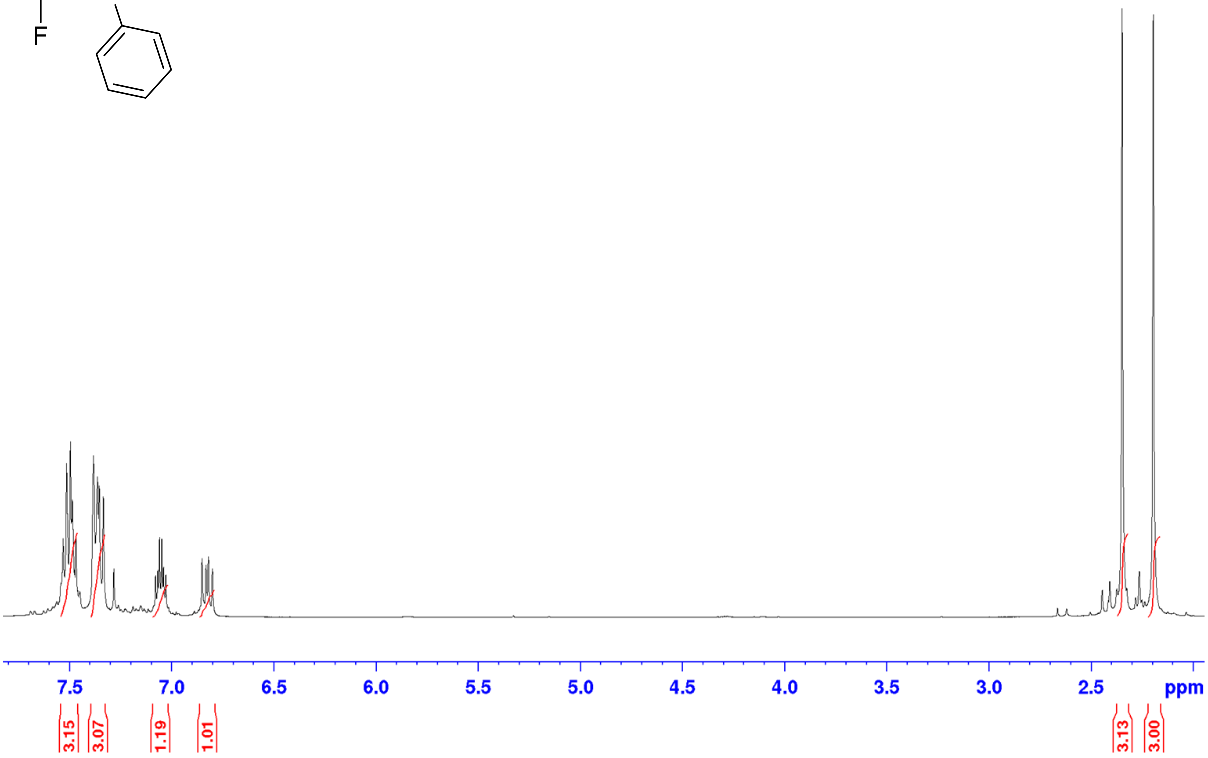

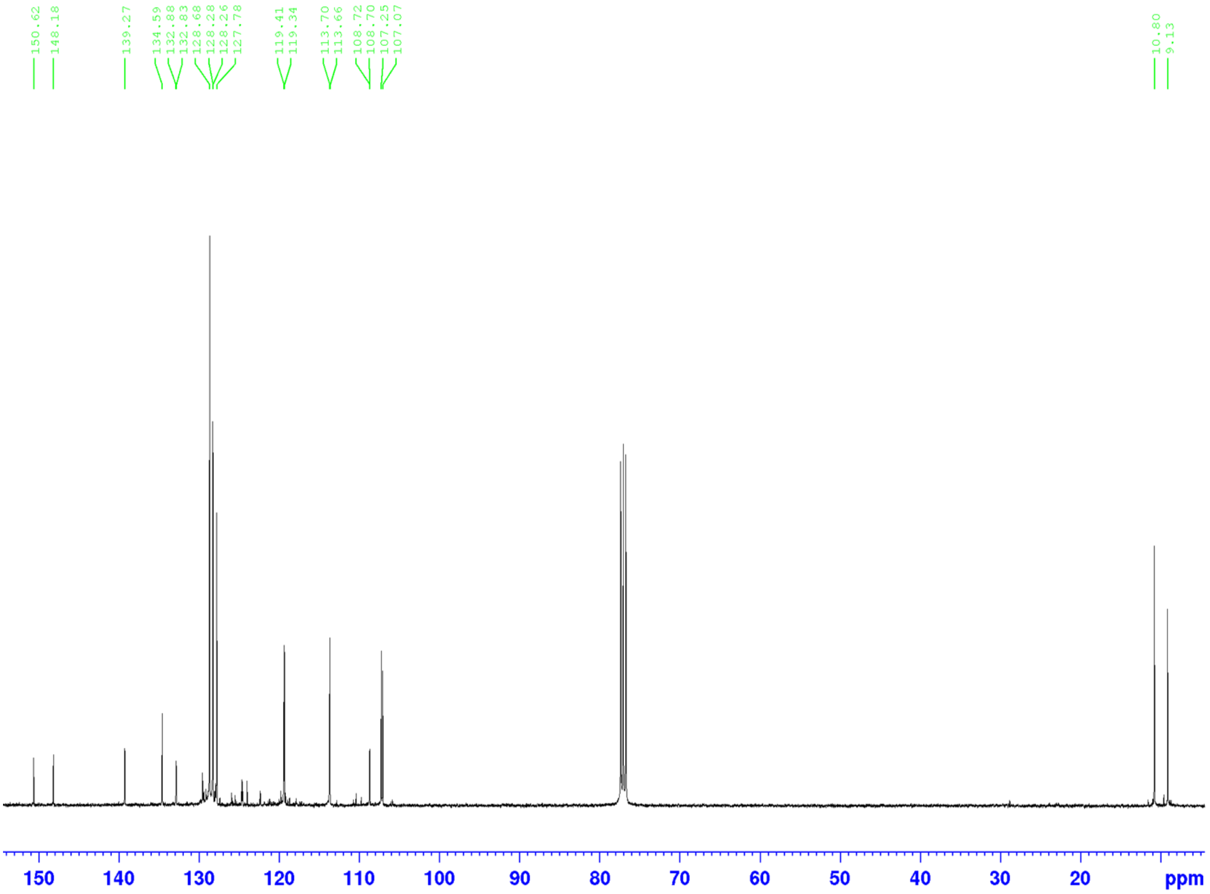

^{1}H and ^{13}C spectra for **6g**

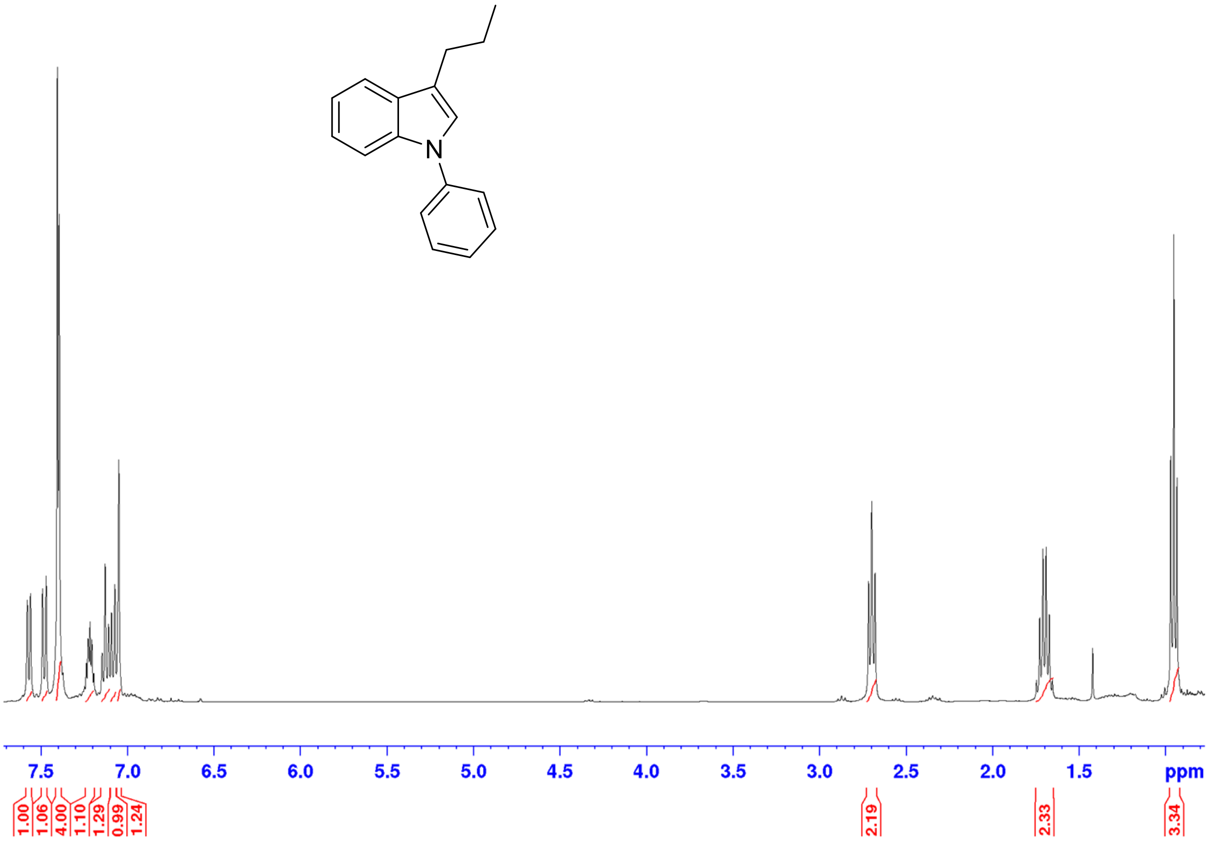

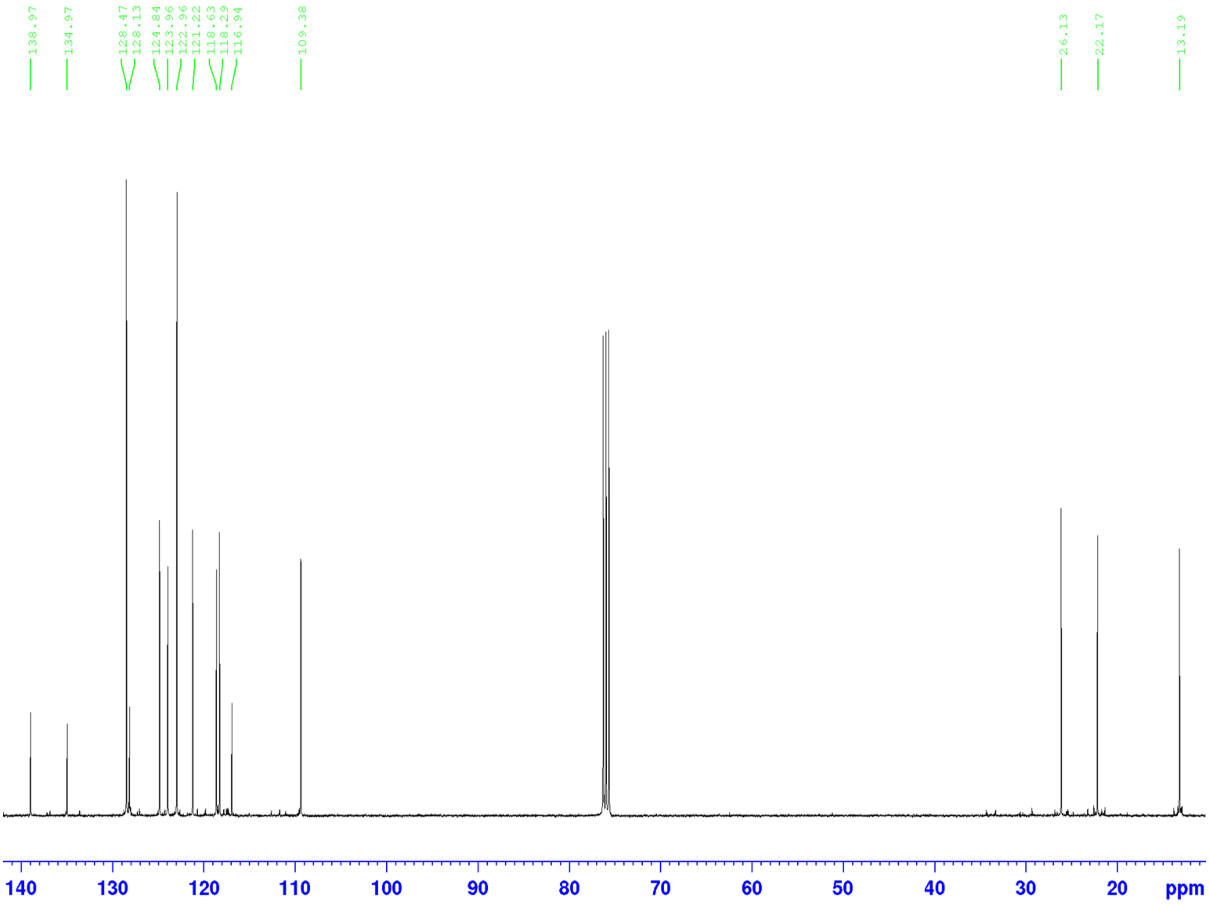

^{1}H and ^{13}C spectra for **6h**

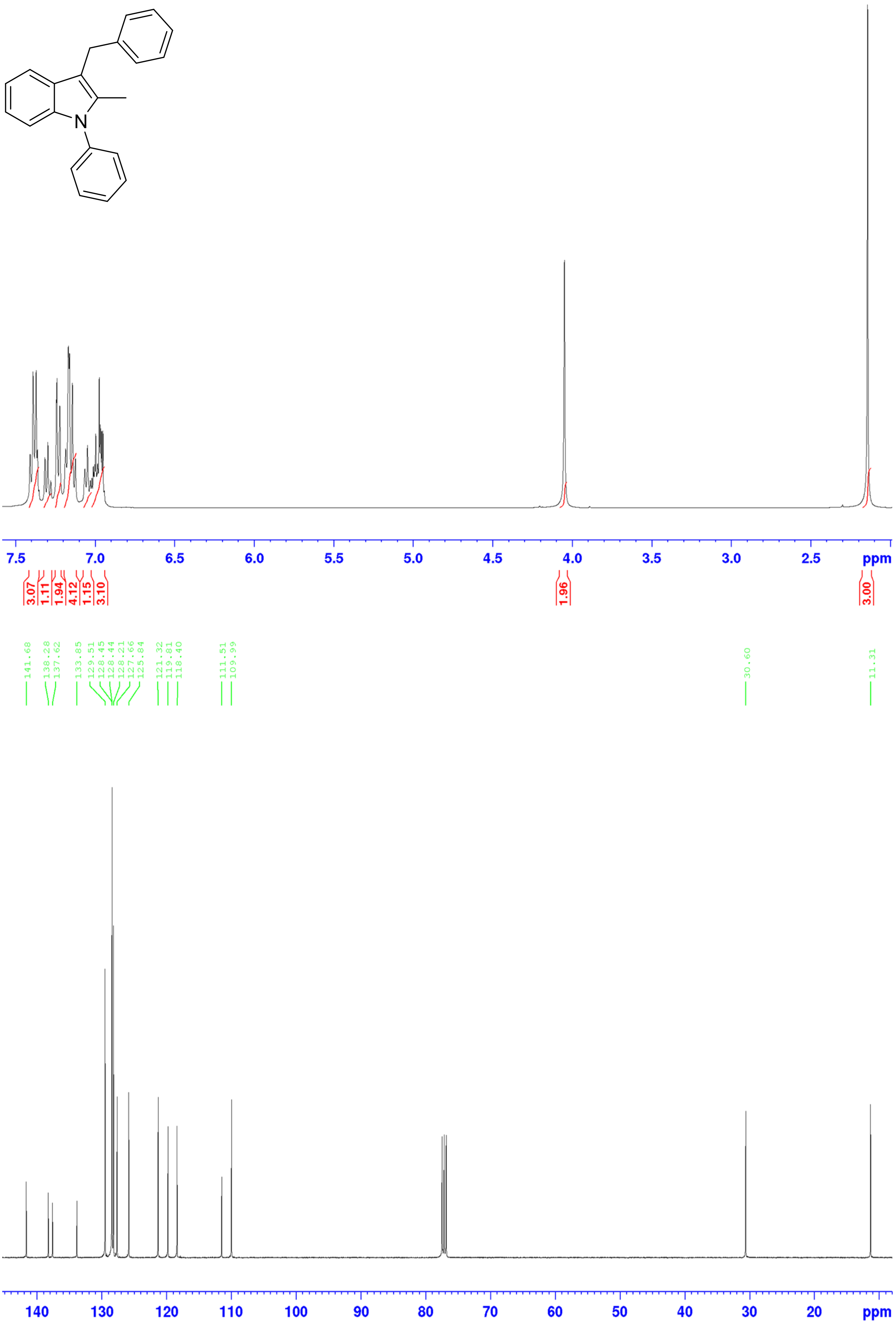

^{1}H and ^{13}C spectra for **6i**

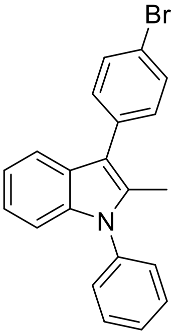

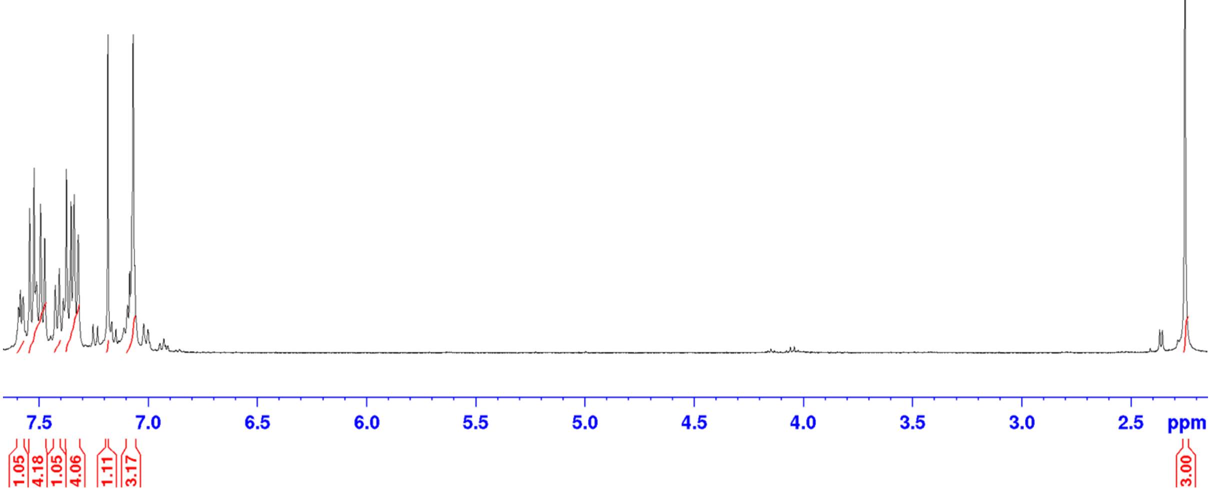

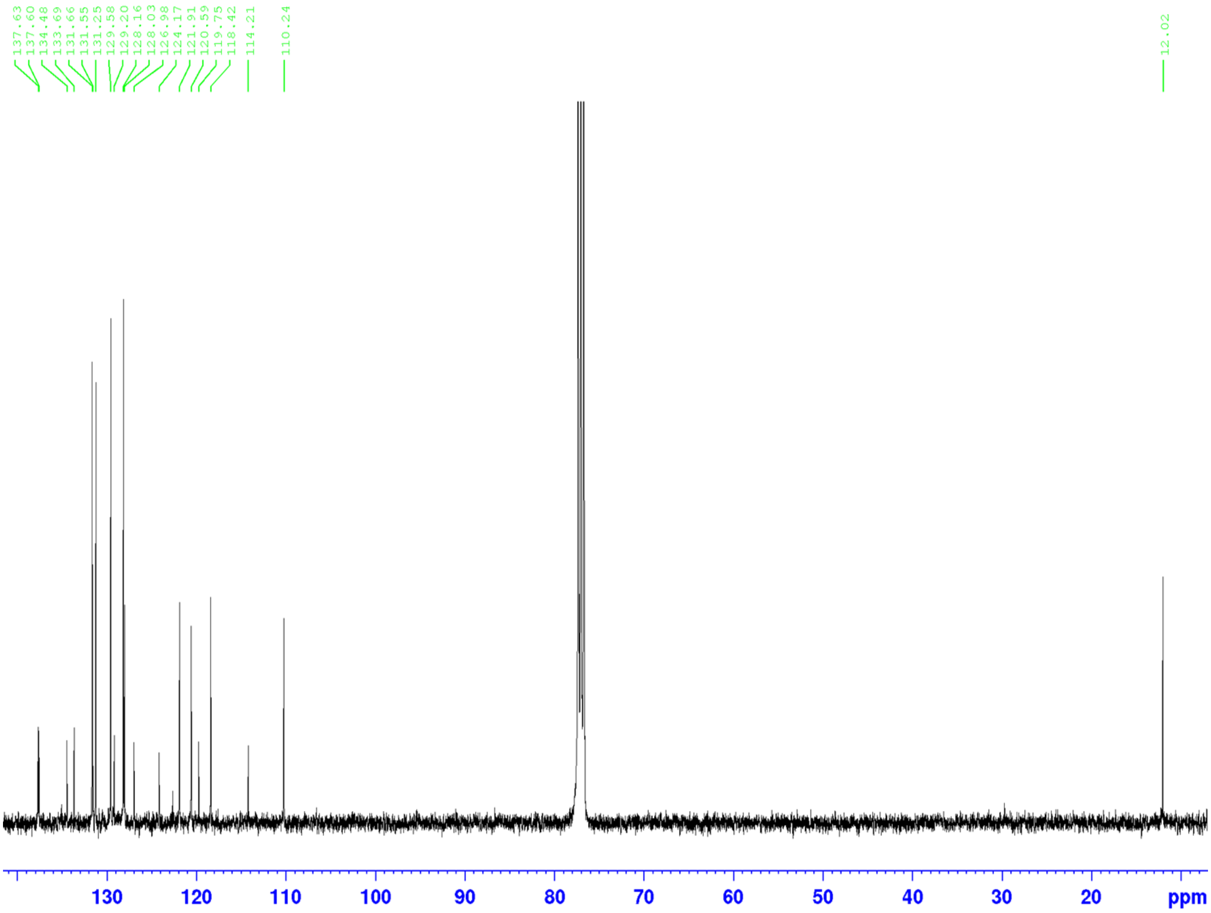

^{1}H and ^{13}C spectra for **6k**

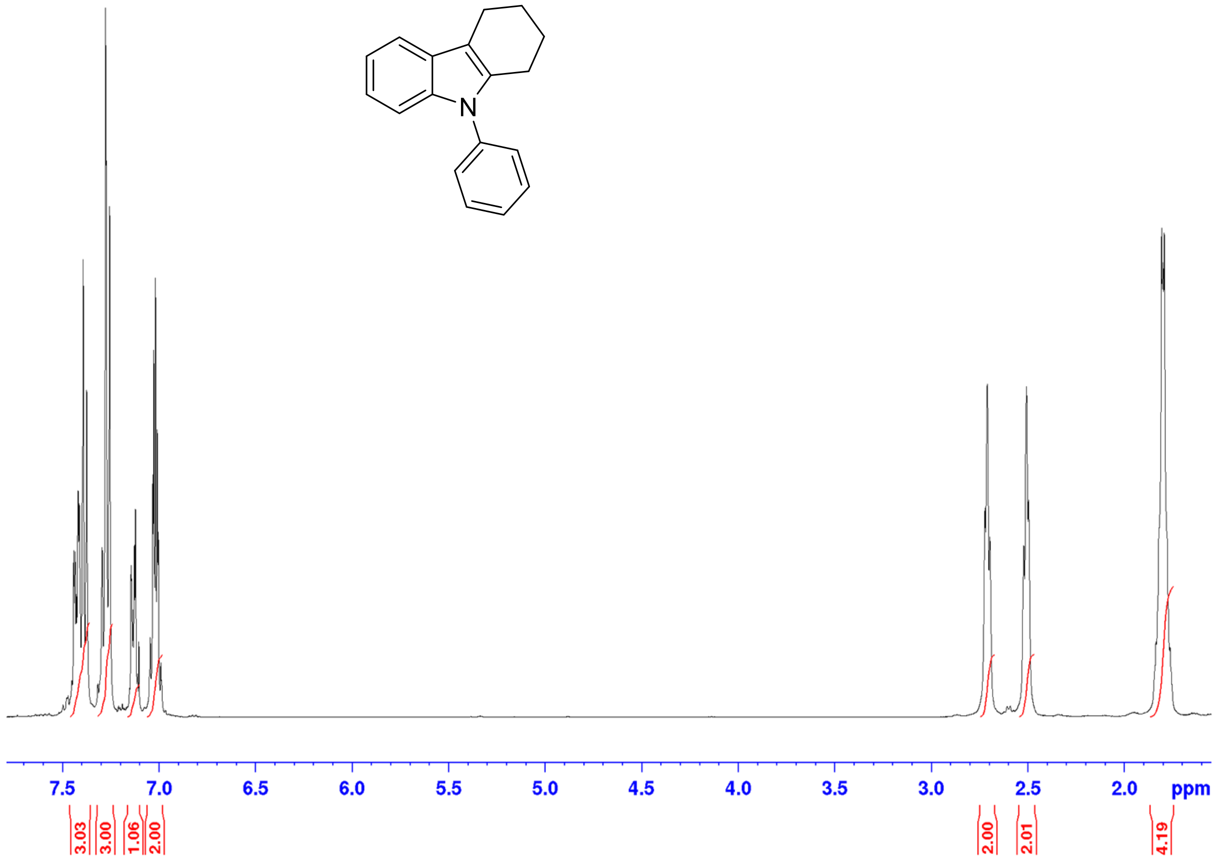

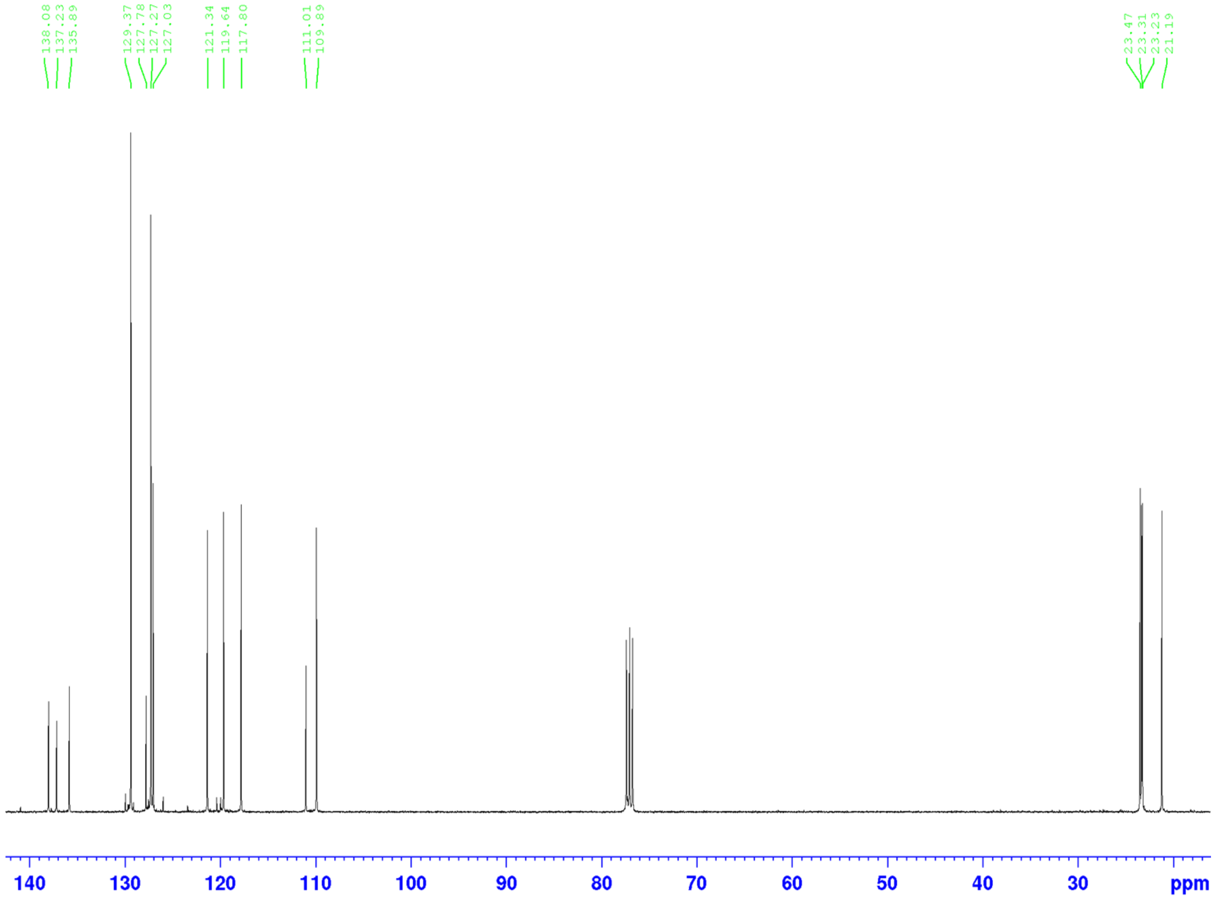

^{1}H and ^{13}C spectra for **6l**

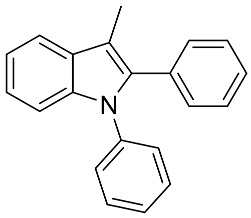

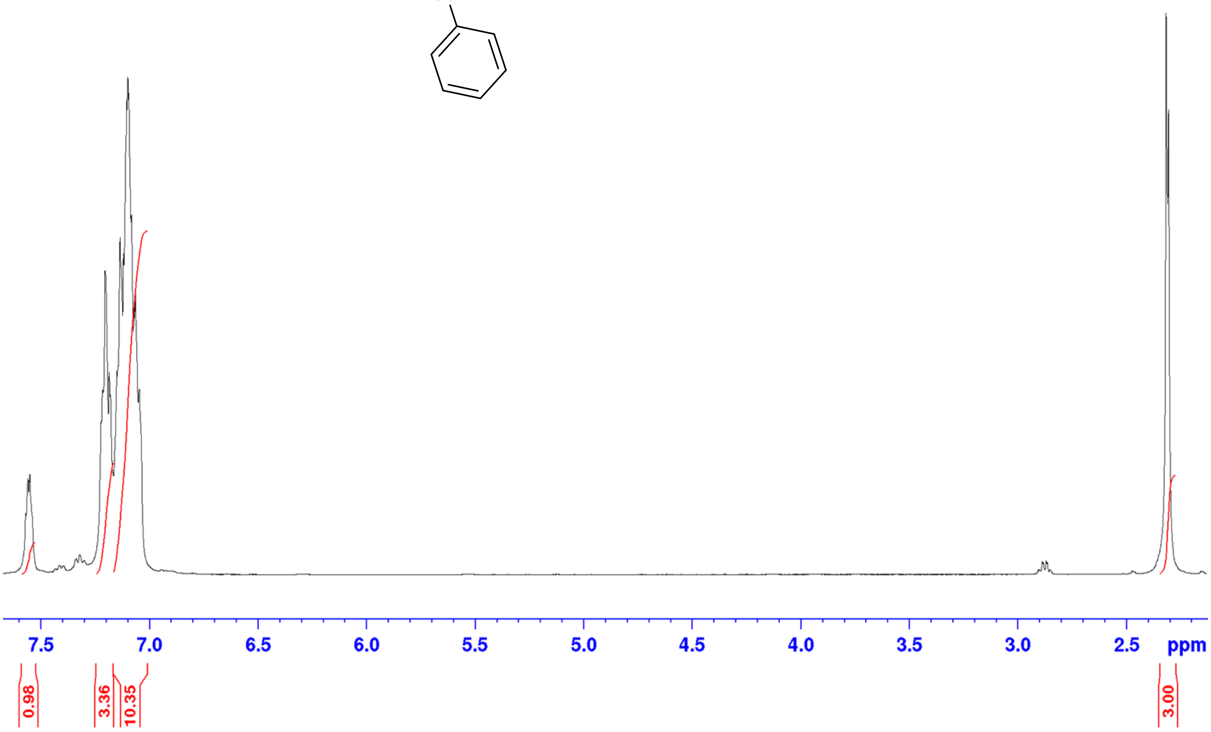

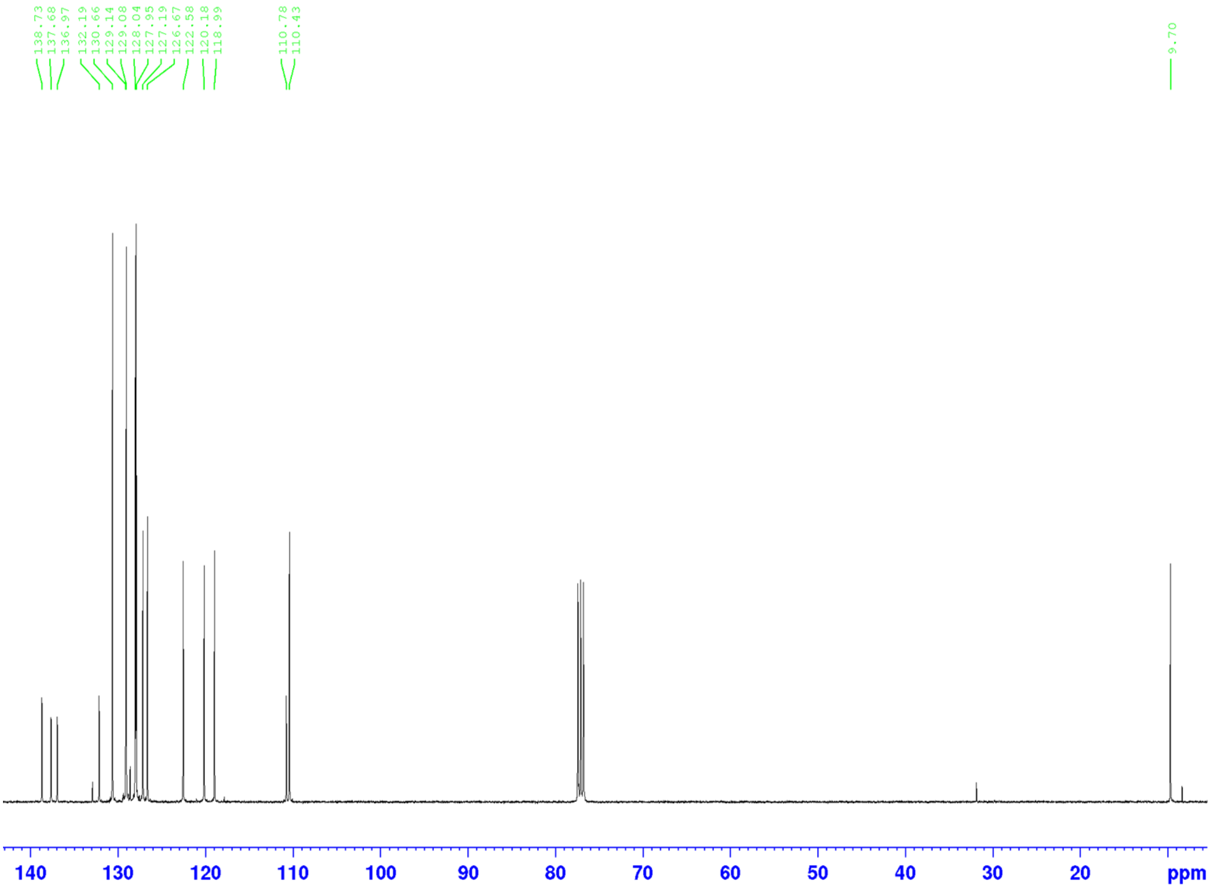

^{1}H and ^{13}C spectra for **6m**

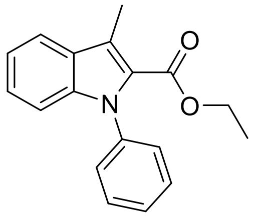

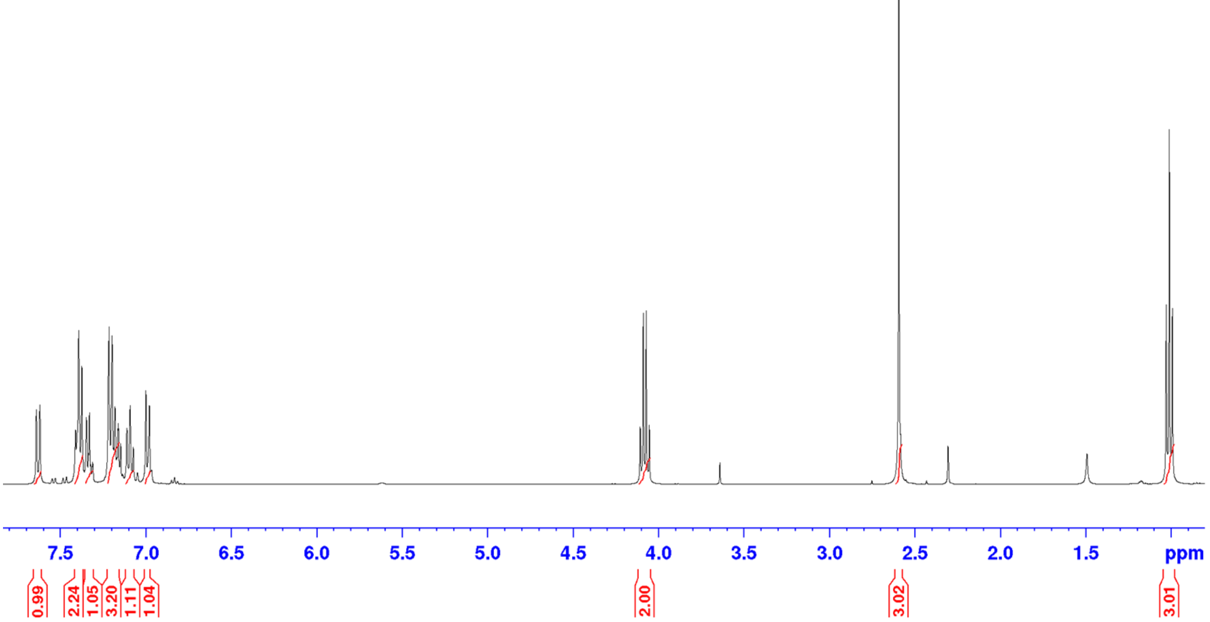

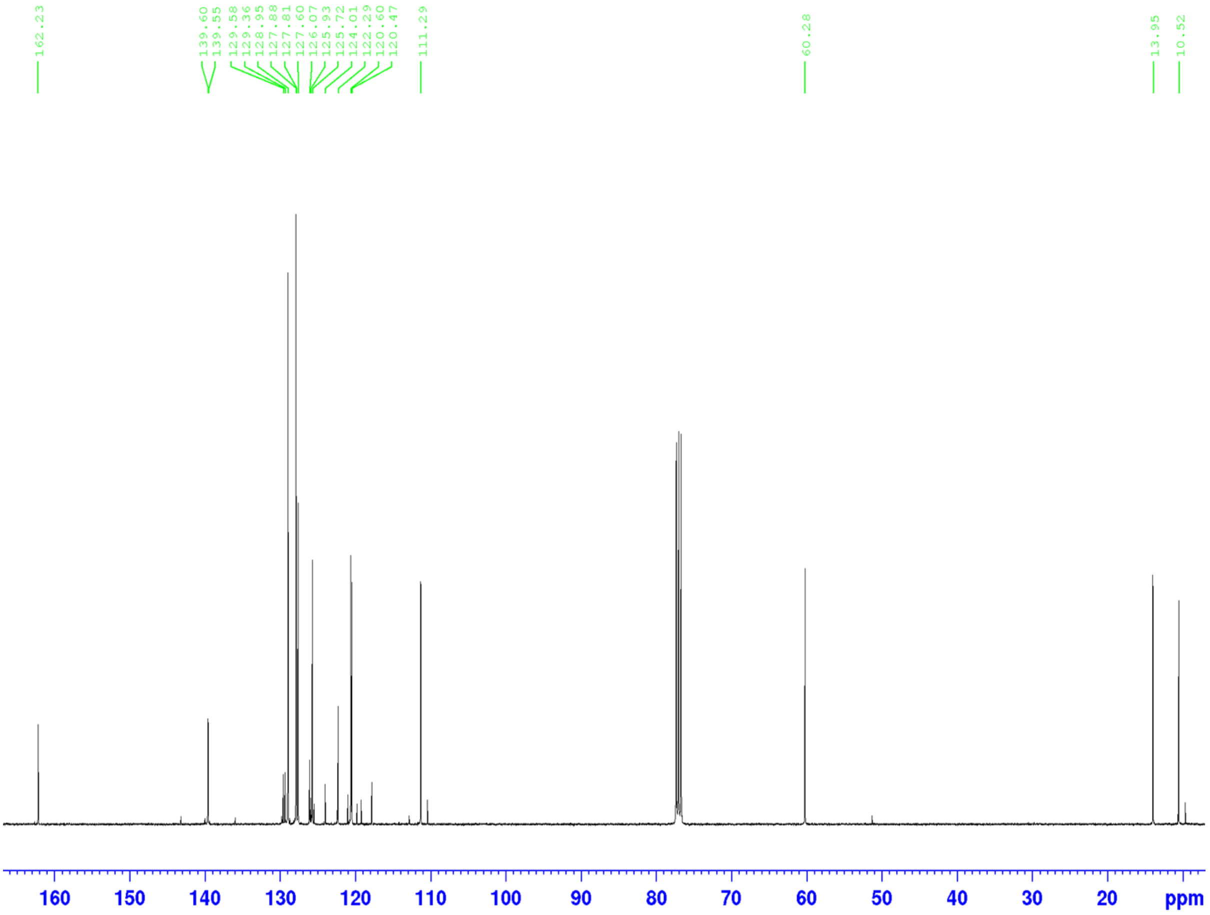

^{1}H and ^{13}C spectra for **6n**

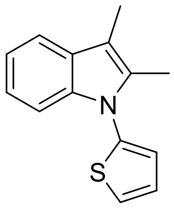

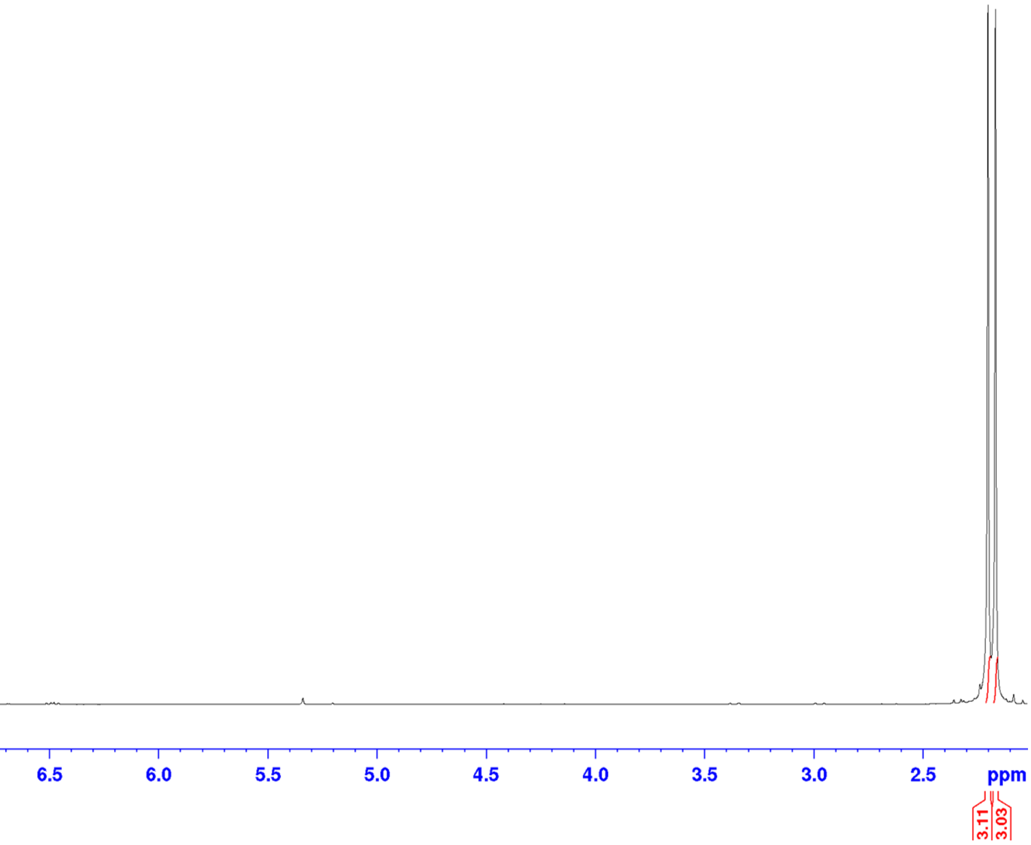

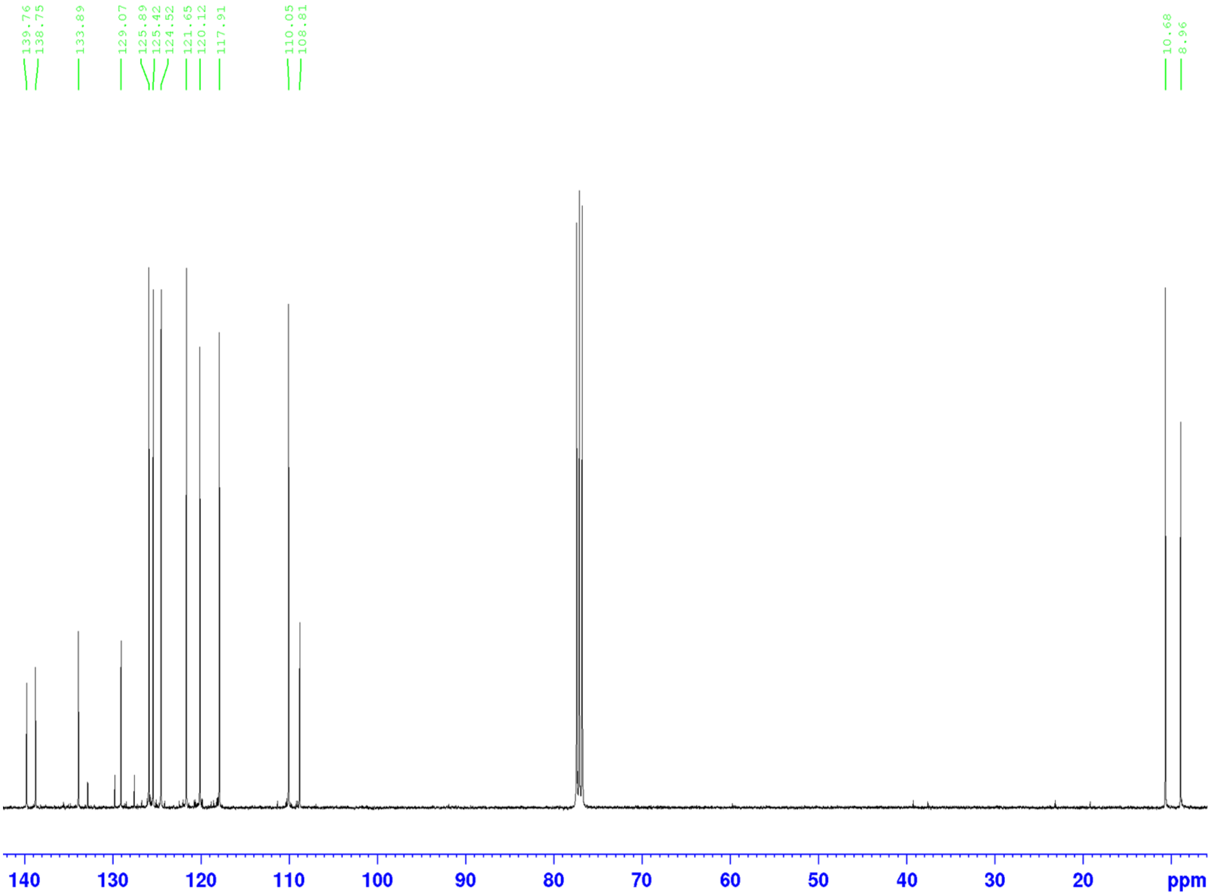

^{1}H and ^{13}C spectra for **6o**

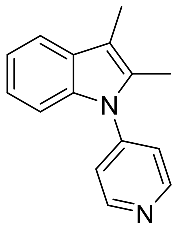

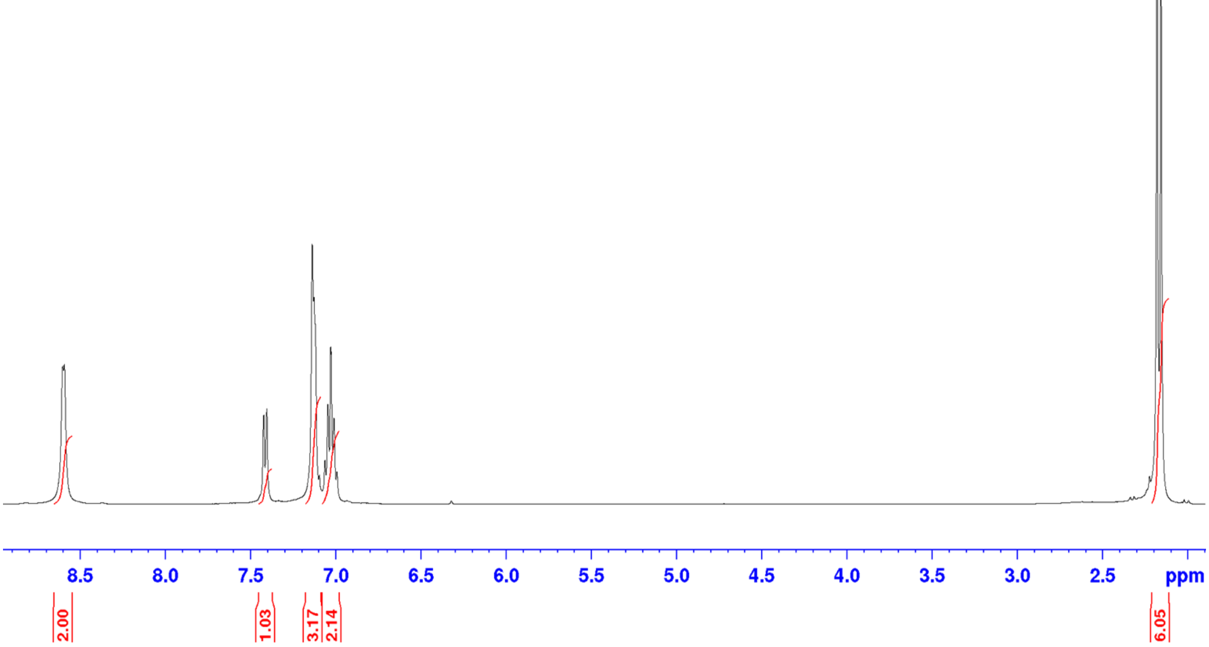

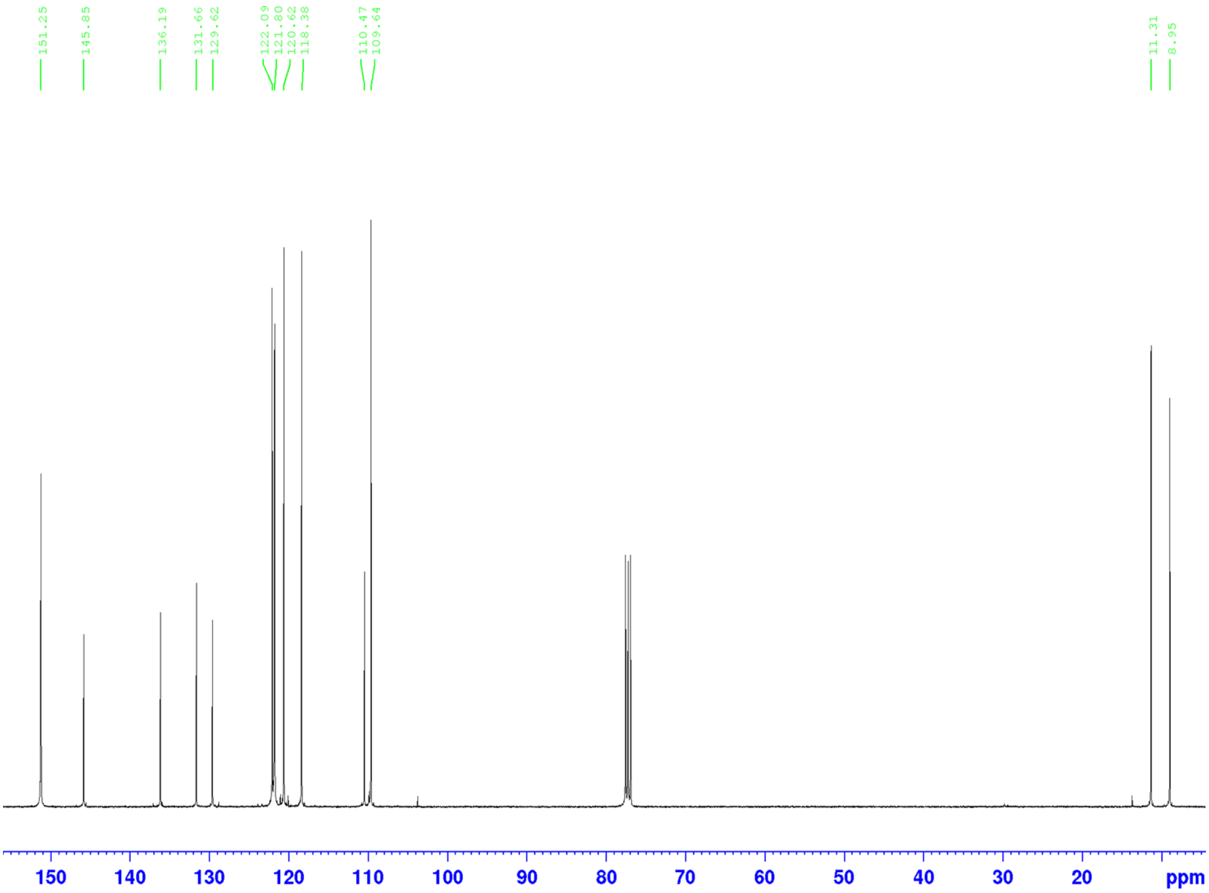

^{1}H and ^{13}C spectra for **6q**

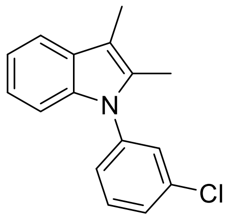

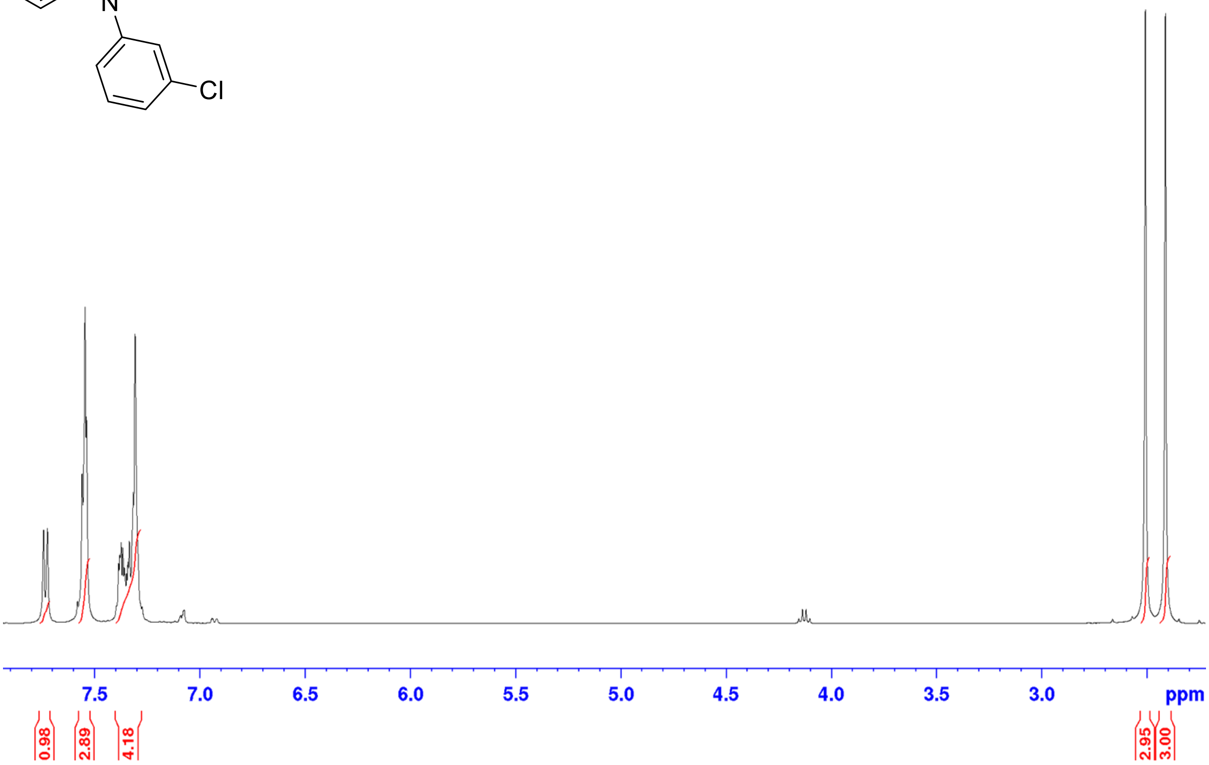

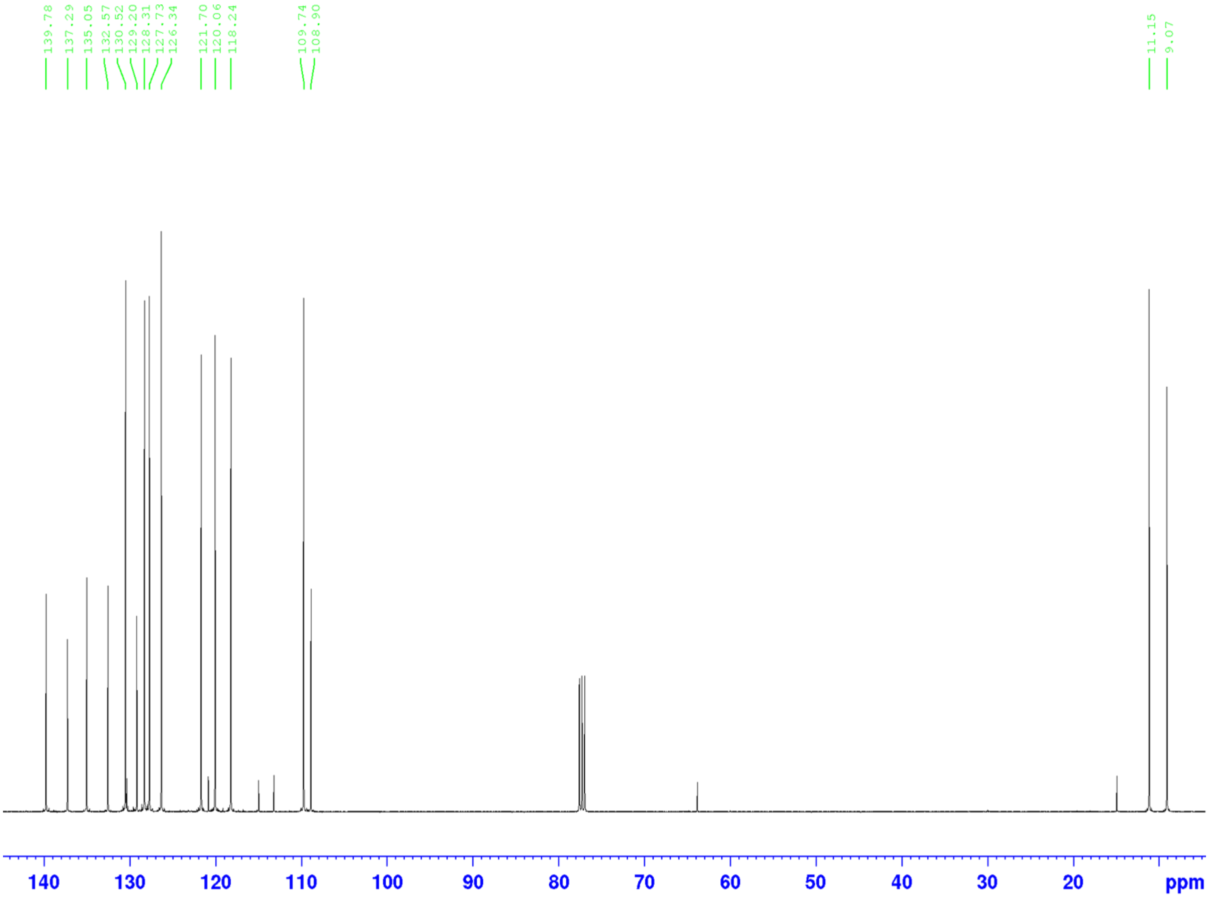

^{1}H and ^{13}C spectra for **6r**

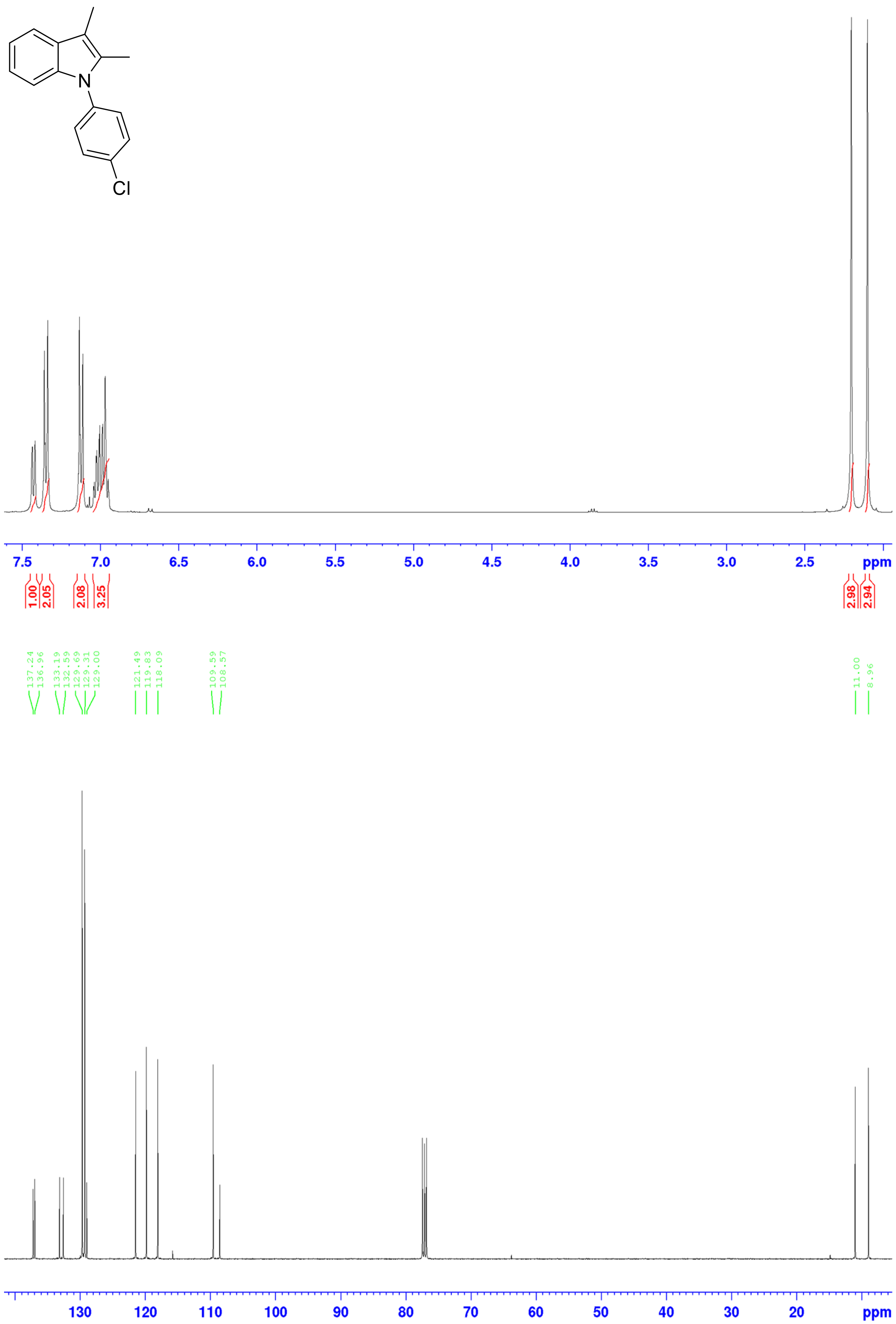

^{1}H and ^{13}C spectra for **6s**

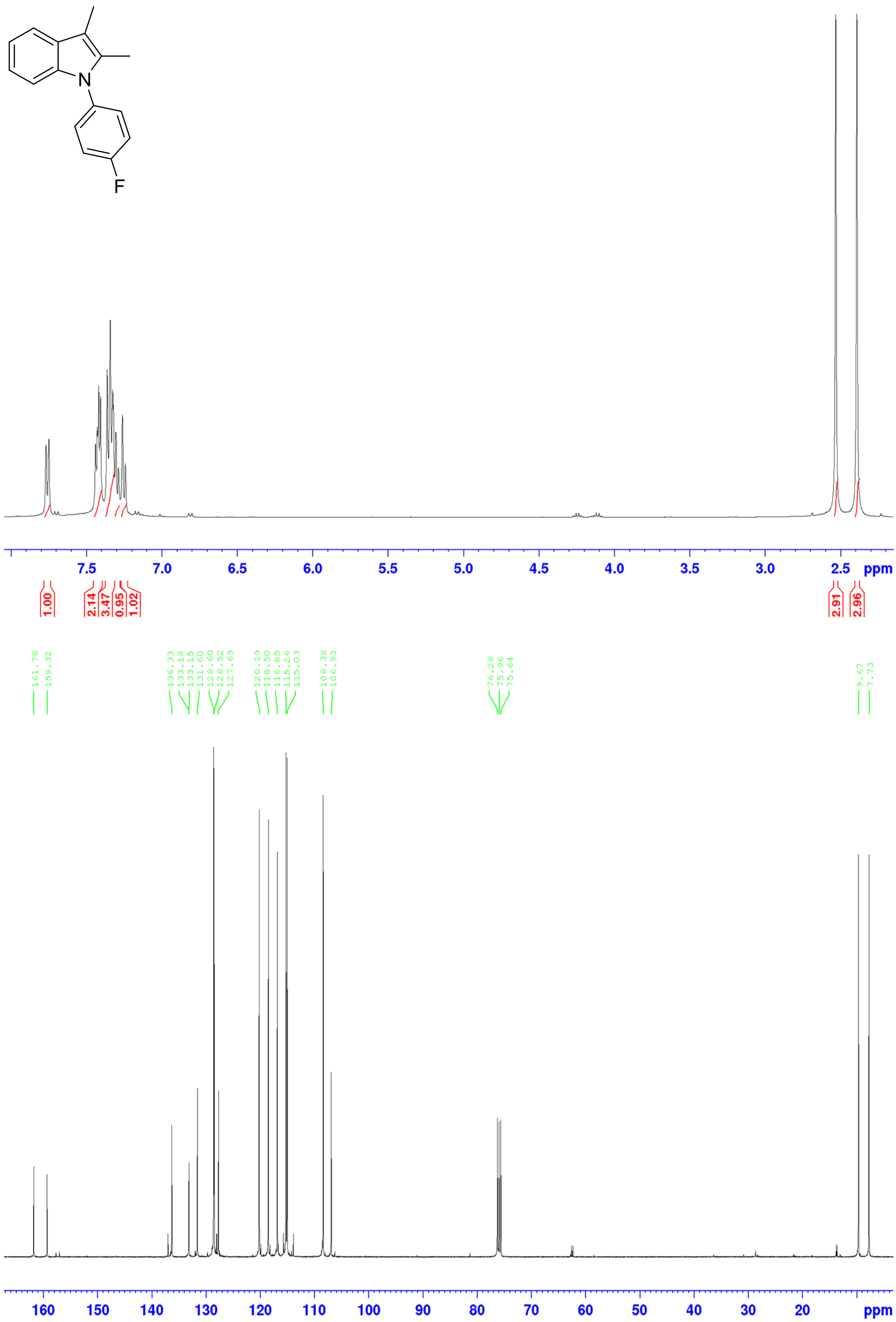

^{1}H and ^{13}C spectra for **6t**

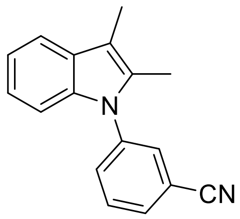

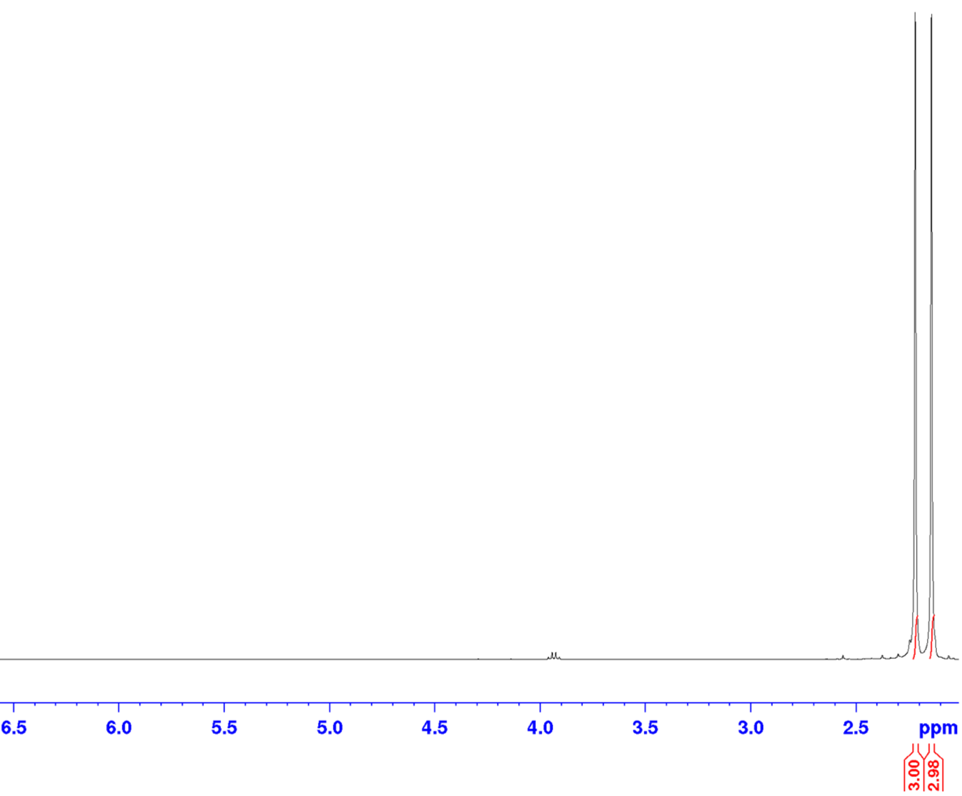

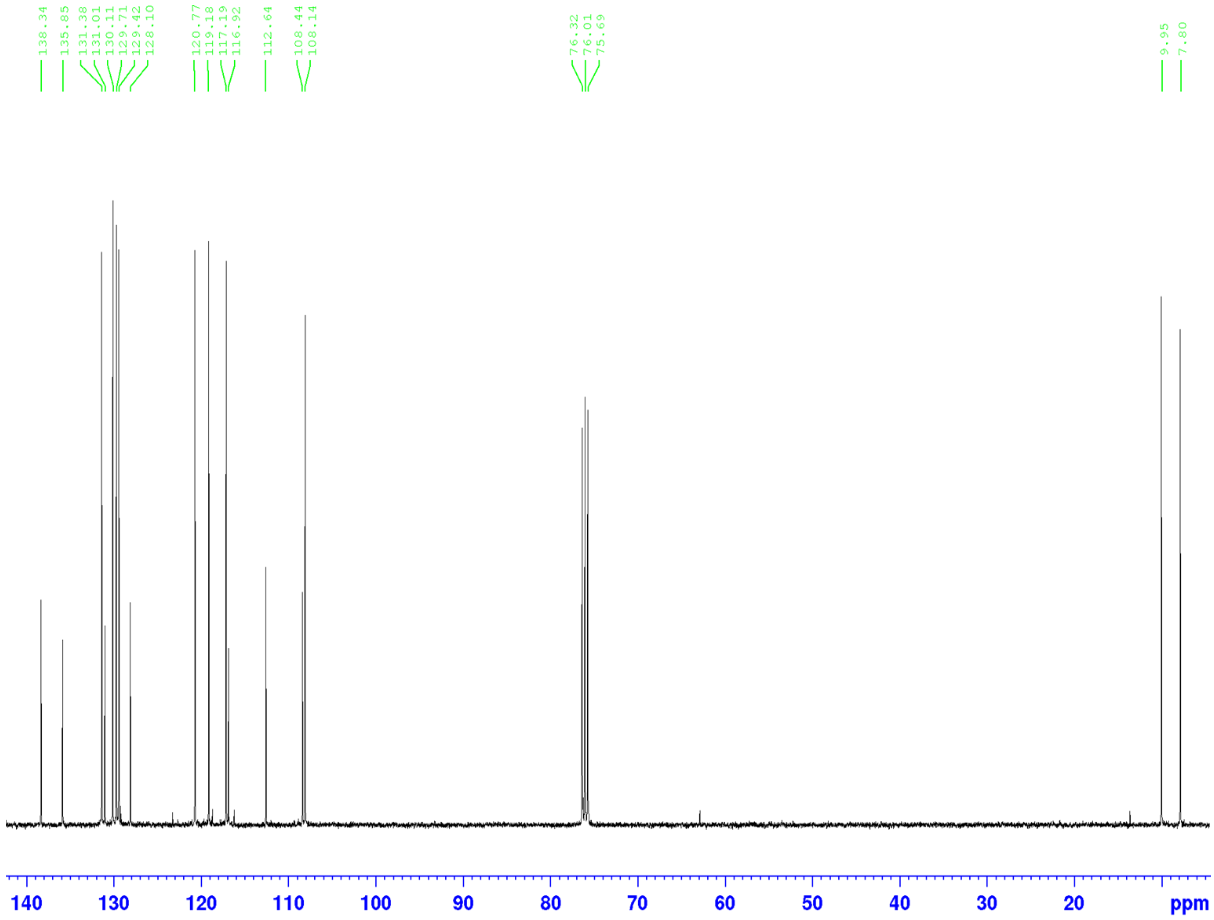

^{1}H and ^{13}C spectra for **6u**

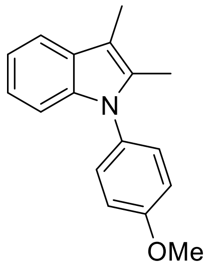

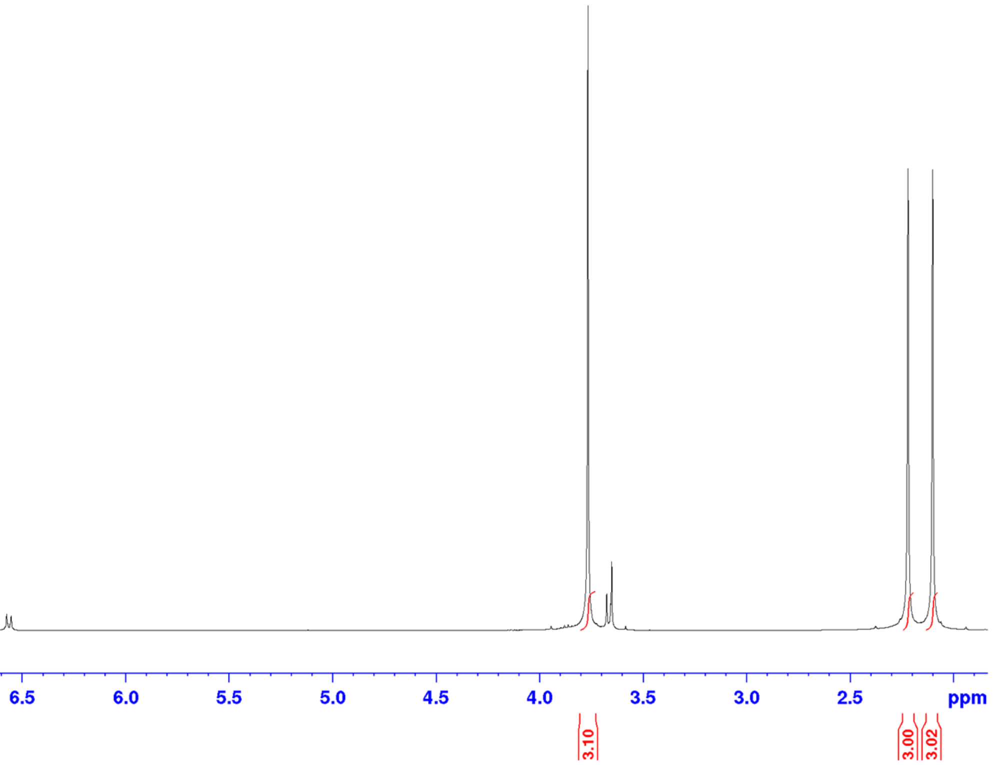

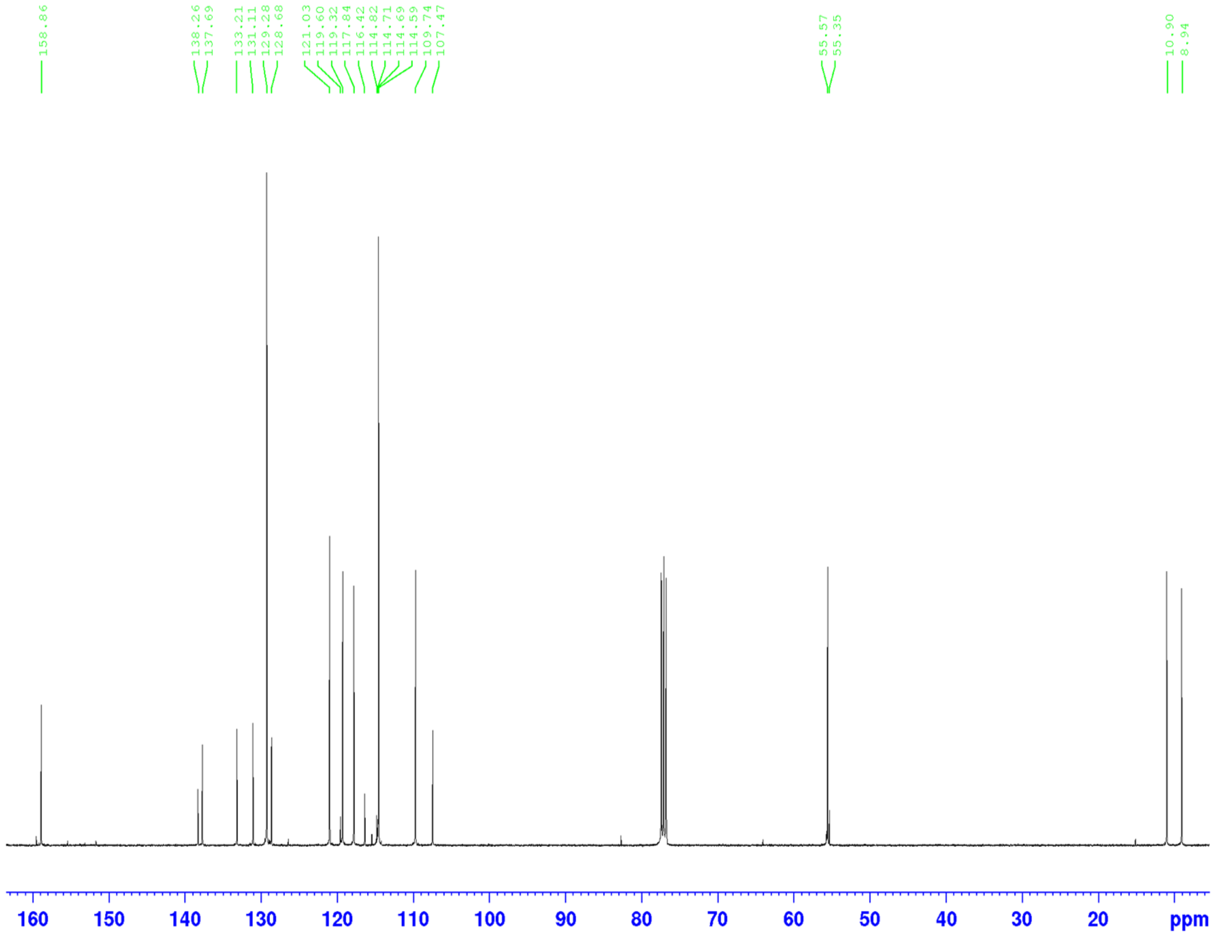

4. References for supporting information

1. G. P. Tokmakov and I. I. Grandberg, *Tetrahedron*, 1995, **51**, 2091–2098.

2. T. Gehrmann, S. A. Scholl, J. L. Fillol, H. Wadepohl and L. H. Gade, *Chem. Eur. J.*, 2012, **18**, 3925–3941.

3. A. Porcheddu, M. G. Mura, L. De Luca, M. Pizzetti and M. Taddei, *Org. Lett.*, 2012, **14**, 6112–6115.

4. J. Barluenga, A. Jiménez-Aquino, F. Aznar and C. Valdés, *J. Am. Chem. Soc.*, 2009, **131**, 4031–4041.

5. G. A. Chesnokov, A. A. Ageshina, M. A. Topchiy, M. S. Nechaev and A. F. Asachenko, *Eur. J. Org. Chem.*, 2019, 4844–4854.

6. S. Deng, M. Wu, E. D. Turtle, W. Ho, M. P. Arend, H. Cheng and L. A. Flippin, *US Patent*, 2008, US2008004309A1.

7. A. Bunescu, C. Piemontesi, Q. Wang and J. Zhu, *Chem. Commun.*, 2013, **49**, 10284–10286.
